# Humans Use a Temporally Local Code for Vibrotactile Perception

**DOI:** 10.1523/ENEURO.0263-21.2021

**Published:** 2021-11-03

**Authors:** Arindam Bhattacharjee, Christoph Braun, Cornelius Schwarz

**Affiliations:** 1 Werner Reichardt Center for Integrative Neuroscience, Systems Neuroscience; 2 Department Systemic Neurosciences, Hertie Institute for Clinical Brain Research; 3 MEG Center, Eberhard Karls University, Tübingen 72076, Germany; 4 Department of Psychology and Cognitive Science, University of Trento, Rovereto TN 38068, Italy; 5 Center for Mind/Brain Sciences, University of Trento, Matarello TN 38123, Italy

**Keywords:** computer simulation, human, psychophysics, tactile coding, vibrotactile

## Abstract

Sensory environments are commonly characterized by specific physical features, which sensory systems might exploit using dedicated processing mechanisms. In the tactile sense, one such characteristic feature is frictional movement, which gives rise to short-lasting (<10 ms), information-carrying integument vibrations. Rather than generic integrative encoding (i.e., averaging or spectral analysis capturing the “intensity” and “best frequency”), the tactile system might benefit from, what we call a “temporally local” coding scheme that instantaneously detects and analyzes shapes of these short-lasting features. Here, by employing analytic psychophysical measurements, we tested whether the prerequisite of temporally local coding exists in the human tactile system. We employed pulsatile skin indentations at the fingertip that allowed us to trade manipulation of local pulse shape against changes in global intensity and frequency, achieved by adding pulses of the same shape. We found that manipulation of local pulse shape has strong effects on psychophysical performance, arguing for the notion that humans implement a temporally local coding scheme for perceptual decisions. As we found distinct differences in performance using different kinematic layouts of pulses, we inquired whether temporally local coding is tuned to a unique kinematic variable. This was not the case, since we observed different preferred kinematic variables in different ranges of pulse shapes. Using an established encoding model for primary afferences and indentation stimuli, we were able to demonstrate that the found kinematic preferences in human performance, may well be explained by the response characteristics of Pacinian corpuscles (PCs), a class of human tactile primary afferents.

## Significance Statement

Sensory systems may exploit specific physical features characteristic for the sensory signal at hand. Recent evidence from rodents showed that very short (“local”) signatures in vibrotactile signals may carry significant amount of texture information; this motivated us to investigate whether the human tactile system is able to exploit information carried by short-duration pulse shapes. We demonstrate that the humans are indeed able to extract information from local pulse shapes. We also present evidence that local cues may be perceptually more effective than the classic “global” variables-intensity and frequency. We tag this encoding scheme as “temporally local code” in analogy to the “spatially local code,” long assumed to be implemented in the visual system.

## Introduction

**Figure 1.**
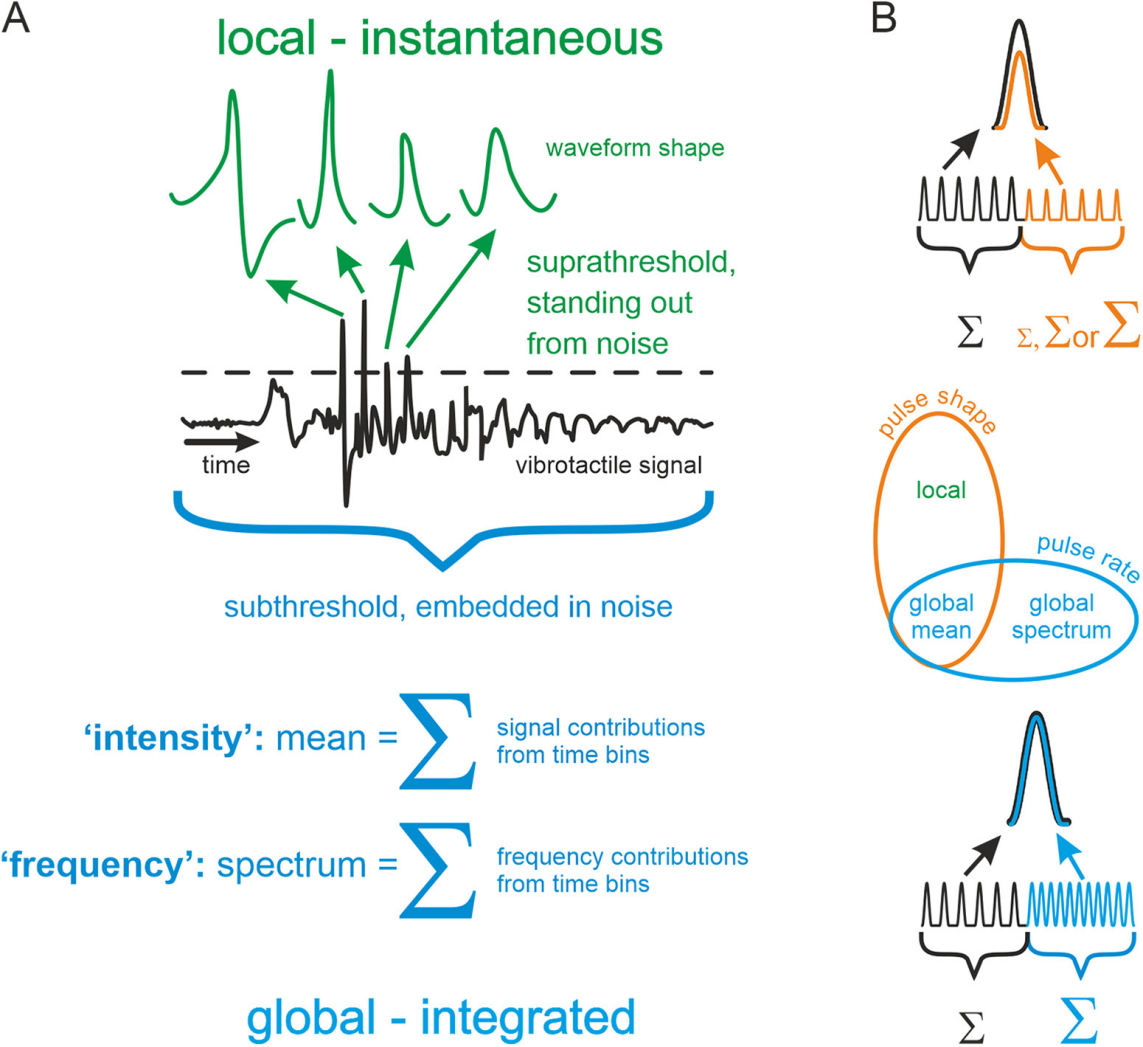
Pictorial description of the terms local/global coding and stimulus manipulations used in this study. ***A***, Illustration of the difference between local and global in the time domain of an arbitrary vibrotactile signal (plotted only for explanatory purposes, this study only used pulsatile stimuli). The defining principle of local coding is the instantaneous extraction of short features from the signal; for example, a simple decoding mechanism could be thresholding (dashed line), but presumably other more advanced change detection algorithms are also feasible. Global coding, on the contrary, assumes that a low fidelity signal, embedded in noise is extracted by integration (summing or averaging) – either signal (intensity) or frequency contributions (frequency) per unit time. ***B***, Experimental realization with pulsatile stimuli in the present study. Participants received pulsatile stimuli starting with a reference stimulus (black) followed seamlessly by a comparison stimulus (color; in 50% of the stimuli, there was no change; data not shown). For clarity, only a cut-out of the stimulus is shown around the change. Above the pulse train, magnified single pulse waveforms are shown; below the pulse train, the change in intensity, as indicated by the size of the Σ sign, is shown. The change from reference to comparison stimulus was done by manipulating either pulse shape (orange) or pulse rate (blue). Center schematic, These manipulations did not map in unequivocal ways on to the coding schemes shown in ***A***, i.e., varying shape (orange oval) affects the local amplitude and width, but also the global intensity, while varying rate (blue oval) affects intensity (mean) as well as rate (spectrum). Note that the effect of rate increments is always an intensity increment. Changes in pulse shape, on the other hand, would increase or decrease intensity depending on the formulation of intensity (e.g., as mean velocity, mean acceleration, etc.; compare possible intensity formulations in Fig. 2).

Sensory systems evolved in contexts that are characterized by physical features of incoming signals. To overcome any constraints that limit the usage of sensory signals, there exists dedicated, non-generic sensory mechanisms that extract special features to accomplish sensory processing. One well-known and illustrative example is local contour processing in mammalian vision. The feature could certainly be captured to some degree by generic approaches, globally integrating across the visual field, as has been suggested (Campbell and Maffei, 1974). However, today it is commonly thought that special purpose local decoding strategies are at work, for instance, small receptive fields, tuned to orientation and short line segments (Hubel and Wiesel, 1968). Similar ideas recently have attracted heightened attention in the tactile field, as frictional movements are increasingly recognized as a physical constraint specific for fine texture discrimination (Hollins and Risner, 2000; Ritt et al., 2008; Wolfe et al., 2008; Jadhav et al., 2009; Arabzadeh et al., 2014; Delhaye et al., 2014; Barrea et al., 2018; Oladazimi et al., 2018). Frictional movements impose ultra-short-lasting vibrational events of tactile sensors, often referred to as stick-slip events (“slips”; Schwarz, 2016). Rodent whiskers convey information about touched textures, encoded in the kinematic outline of slips, which are easily detected as they stand out well against the noise (Wolfe et al., 2008). Importantly, information about texture in short slip waveforms appeared to be even superior to the one conveyed by the more generic analyses integrating the signal across time, “intensity” (signal averaging) or “best frequency” (spectral decomposition; Oladazimi et al., 2018). In analogy to the mentioned spatial local and global coding principles in vision, we use the terms local/global to label vibrotactile coding principles in the time domain (i.e., temporally local or temporally global). Figure 1*A* illustrates some core characteristics of temporally local coding. First, it employs short integration windows (for the expected duration of those, see below), and therefore, is (quasi-)instantaneous. Second, it uses detection of outstanding events. The simplest implementation of event detection is thresholding, but there is a host of algorithms that serves the same function in more sophisticated ways (Poor and Hadjiliadis, 2008). These two core characteristics enable temporally local coding schemes to discriminate the shape of short-lasting events. In contrast, temporally global coding uses summing of either signal or frequency contributions across a comparably longer stimulus section. This is not to say that temporally global coding neglects local event shapes, but it is affected by those only in more indirect ways (Fig. 1*A*; Schwarz, 2016).

Here, we set out to investigate whether humans can use temporally local shapes of vibrotactile waveforms for perception. To be able to compare with classical experimental (LaMotte and Mountcastle, 1975; Johansson, 1978) and modeling work (Saal et al., 2017), we report about perception of pulsatile indentations of the fingertip skin. Indentation, at the first glance, seems not related as much to frictional movements, which often are thought to relate to tangential sideways movement of the fingertip. There can be no doubt, however, that even during tangential movement across surfaces with asperities, the skin is engaged in vertical (indenting) directions as well. The pulsatile nature of our stimuli allowed us to precisely manipulate local shape of single pulses and trade their effect on temporally global coding variables, like intensity and frequency, which rely on cross-pulse integration, as was done before in whiskers (Waiblinger et al., 2015a). The analytic nature of pulsatile stimuli dictates the time criterion differentiating local versus global coding: we presented pulses at rates of 90 and 30 Hz, the interpulse duration of which confined local coding schemes to windows shorter than 11 and 33 ms, respectively. The presentation of these two ranges of pulse rates were firstly motivated to demonstrate possible local coding in two encoding ranges classically defined using long sinusoidal stimuli, the “flutter range” (20–80 Hz), thought to be captured largely by rapidly adapting skin receptors of type 1 and the “Pacinian range” (larger than 150 Hz), thought to be encoded solely by rapidly adapting receptors of type 2 (Talbot et al., 1968; Bolanowski et al., 1988). Second, both windows are compatible with biomechanical measurements of slips in skin and whiskers (Johansson and Westling, 1987; Wolfe et al., 2008; Delhaye et al., 2014; Oladazimi et al., 2018), integration times of primary afferents (Johansson and Birznieks, 2004; Chagas et al., 2013; Pruszynski and Johansson, 2014; Birznieks and Vickery, 2017; Laturnus et al., 2021), and behavior (Gerdjikov et al., 2010, 2018; Stüttgen and Schwarz, 2010; Waiblinger et al., 2015a,b).

In this study, we ask whether humans use local pulse shape for detection of changes in pulsatile stimuli. To this end, the tested stimulus systematically traded changes in pulse shape against changes in rate, as illustrated in Figure 1*B*.

## Materials and Methods

### Participants

We recruited a total of 20 neurologically-healthy (self-reported) participants (age: 20–40 years, median 26 years; eight females). Two participants withdrew from the study without providing any reason. Our participant recruitment advertisement discouraged all individuals from participation if they were diagnosed with dyslexia (because it adversely affects tactile acuity (Grant et al., 1999; Laasonen et al., 2001), diabetes (which could result in peripheral neuropathy and action potential conduction delays; Hyllienmark et al., 1995), learning disabilities, nervous system disorders, or had any calluses or injury to the left index fingertip (the tested finger). Based on questions modified from the Edinburgh Handedness Inventory (Oldfield, 1971), we classified 19 participants as right handed. The study was approved by our institutional research ethics board; all the participants signed the informed consent form and were paid for their participation in the study.

**Figure 2.**
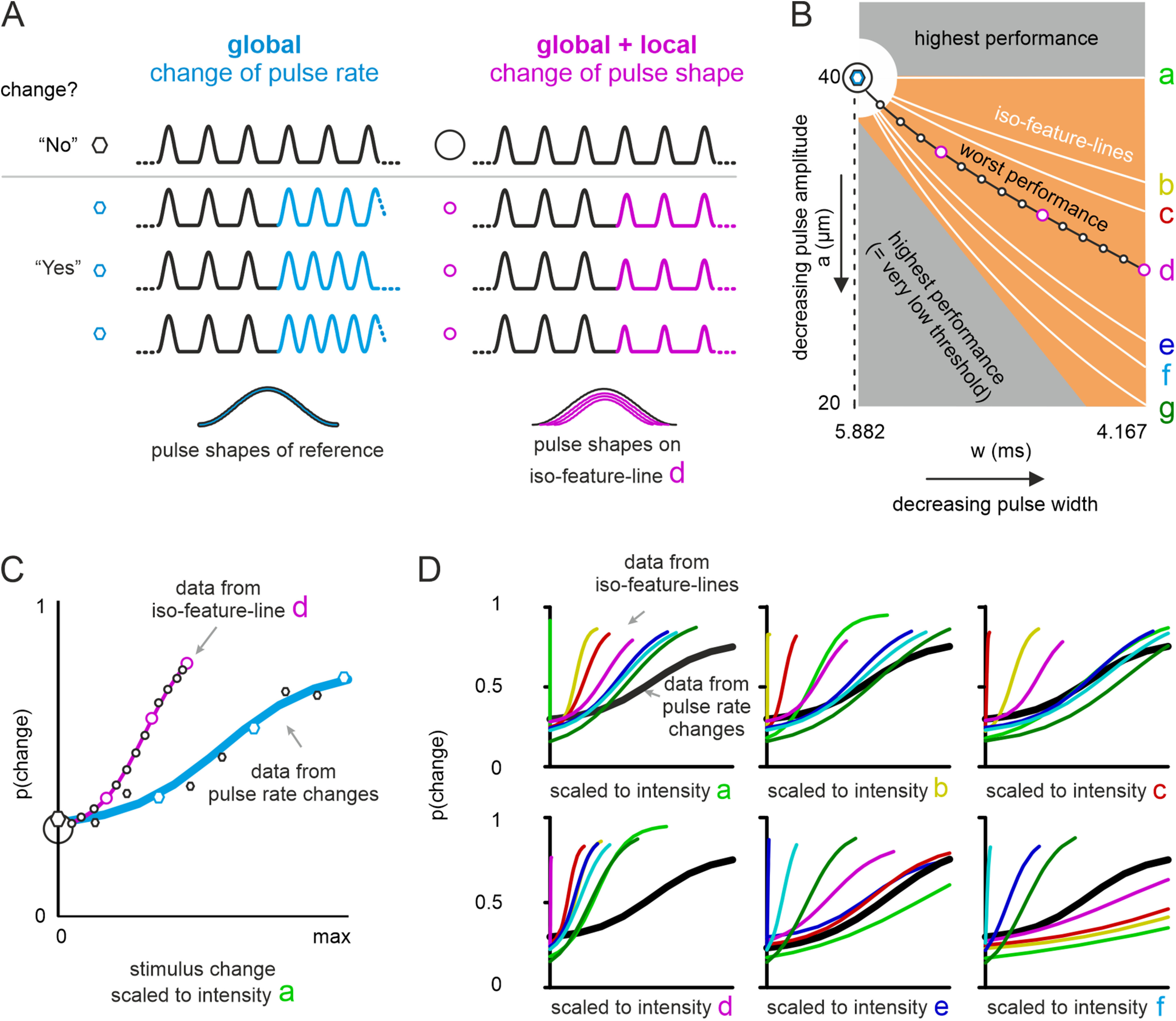
Detection of change using shape and rate manipulation. ***A***, Example stimuli for experiments presenting rate changes (blue) and shape changes (magenta, line d in panel ***B***). Above the gray line are stimuli without change (correct answer: “no”), below some example stimuli with a change (correct answer: “yes”). Bottom, Magnified single pulses from each of the four example stimuli illustrating pulses without (blue) and with (magenta) local shape changes. The stimulus changes identified by the symbols on the left of the traces are also marked in the stimulus space (***B***). ***B***, Stimulus space used to change pulse shape. In these experiments, the pulse rate, i.e., the interpulse interval, was identical for reference and comparison stimuli. The reference stimulus is in the upper left at an amplitude 40 μm and a width of 5.882 ms (large black circle). Changing pulse shapes to points in the space within the gray regions would lead to very good performance. Stimulus changes within the orange area would be detected relatively poorly. The white lines indicate iso-feature-lines holding shape changes that would keep certain formulations of intensity or descriptors of local shape unchanged. Global (intensity) iso-feature-lines: line a, mean velocity; line b, mean squared velocity; line c, mean cubic velocity; line d, mean acceleration; line e, mean squared acceleration; line f, mean cubic acceleration. Local (shape) iso-feature-lines: line a, maximal position; line d, maximal velocity; line g, maximal acceleration. The black and blue hexagons indicate the reference and all comparison stimuli in experiments using rate changes (identical shapes, i.e., no local cue). The symbols on iso-feature-line d point to stimulus changes used for experiments varying shape (stimuli picked from all other iso-feature-lines would use the same pulse widths and the respective amplitudes located on the lines; not shown). ***C***, Psychophysical population data and psychometric curves (logistic fits). All symbols (circles and hexagons refer to the ones shown in panels ***A***, ***B***). Magenta, Pooled data from 10 participants tested on shape changes from line d. Blue, Pooled from nine participants tested on rate changes. ***D***, Data from all seven iso-feature-lines (colors correspond to the letters marking respective iso-feature-lines in ***B***). The thick black line corresponds to the blue line in ***C***. The six graphs replot the same data rescaled to each of the six intensity formulations corresponding to iso-feature-lines a–f (see definitions in the legend of ***B***). The abscissae in panels ***C***, ***D*** are scaled to intensity indicated (range: zero to maximum intensity-change found in the rate-change stimuli).

To identify the stimulus feature that the participants used to perform the perceptual tasks, we required the participants to perform well so that we could generate their psychometric function for each task (see below, Perceptual tasks and psychophysics procedure). If any participant performed poorly on any task (session) and we failed to generate a viable psychometric function for those tasks (passing 50% correct), we ran them again on those specific tasks. The reasoning was that if a critical feature varies (see Fig. 2*B*, orange section, iso-feature-lines a–g) and is absolutely essential to perform the task then eliminating differences in this feature should consistently hinder task performance (see Results, Stimulus space, iso-feature-lines, and terminology). The repetition therefore served as a control whether the inability to perform on the task was because of the assumed lack of variation of a critical feature or because of other reasons. In no case did we detect a systematic inability to perform any of the experiments. We therefore conclude that among the features tested, there is no single feature which is absolutely necessary for performance. One participant was disqualified from the study because the total percent correct for all seven sessions of the pulse shape change experiment (stimulus Range I) was <50%. The data of the disqualified participant and the participants who withdrew from the study are not included in Results. In summary, 10 participants were tested in the pulse shape change (stimulus Range I) experiment, 10 participants were tested in the pulse shape change (stimulus Range II) experiment (three of them also participated in the stimulus Range I experiment); nine of the 10 participants from the stimulus Range I experiment were additionally tested in the pulse rate change experiment as well as the pulse rate and shape (combined) change experiment (Table 1 gives an overview).

**Table 1:** Experimental conditions at a glance

Experimental (change) condition	Pulse			Stimulus levels	Trials*		Participants
	Rate (Hz)	Amplitude (μm)	Width (ms)		Yes	No	
							
Stimulus Range I	Reference stimulus: 90-Hz pulse rate, 5.882-ms pulse width, 40-μm amplitude						
							
**Pulse shape**							
Iso-feature-line a	90	40	5.882–4.167	14	420	420	10
Iso-feature-line b		40–33.6					
Iso-feature-line c		40–31.9					
Iso-feature-line d		40–28.3					
Iso-feature-line e		40–23.9					
Iso-feature-line f		40–22.4					
Iso-feature-line g		40–20					
							
**Pulse rate**	90–135	40	5.882	9	270	270	9
							
**Pulse rate-and-shape**							
Iso-feature-line d’	105	40–28.3	5.882–4.167	14	420	420	9
Iso-feature-line d’’		33.5–24.5					
							
Stimulus Range II	Reference stimulus: 30-Hz pulse rate, 28.571-ms pulse width, 100-μm amplitude						
**Pulse shape**							
Iso-feature-line a	30	100	28.571–15.385	10	300	300	10
Iso-feature-line b		100–74.3					
Iso-feature-line c		100–65.7					
Iso-feature-line d		100–52.8					
Iso-feature-line e		100–40					
Iso-feature-line f		100–35.7					
Iso-feature-line g		100–29.3					

Within the stimulus Range I, the reference stimulus was identical for the pulse shape change, pulse rate change, and pulse rate and shape change experimental conditions; in the stimulus Range II, only pulse shape change conditions were implemented, which all had the same reference stimulus. The values across the table for each condition refers to the comparison stimulus (i.e., the “yes” change trials), which were 500 ms long; in the no-change trials, the reference stimulus is presented for the complete stimulus duration (i.e., 1000 ms). *Total change trials can be calculated by multiplying 30 to the number of stimulus levels; equal number of no-change trials were presented during each experimental condition session.

**Figure 3.**
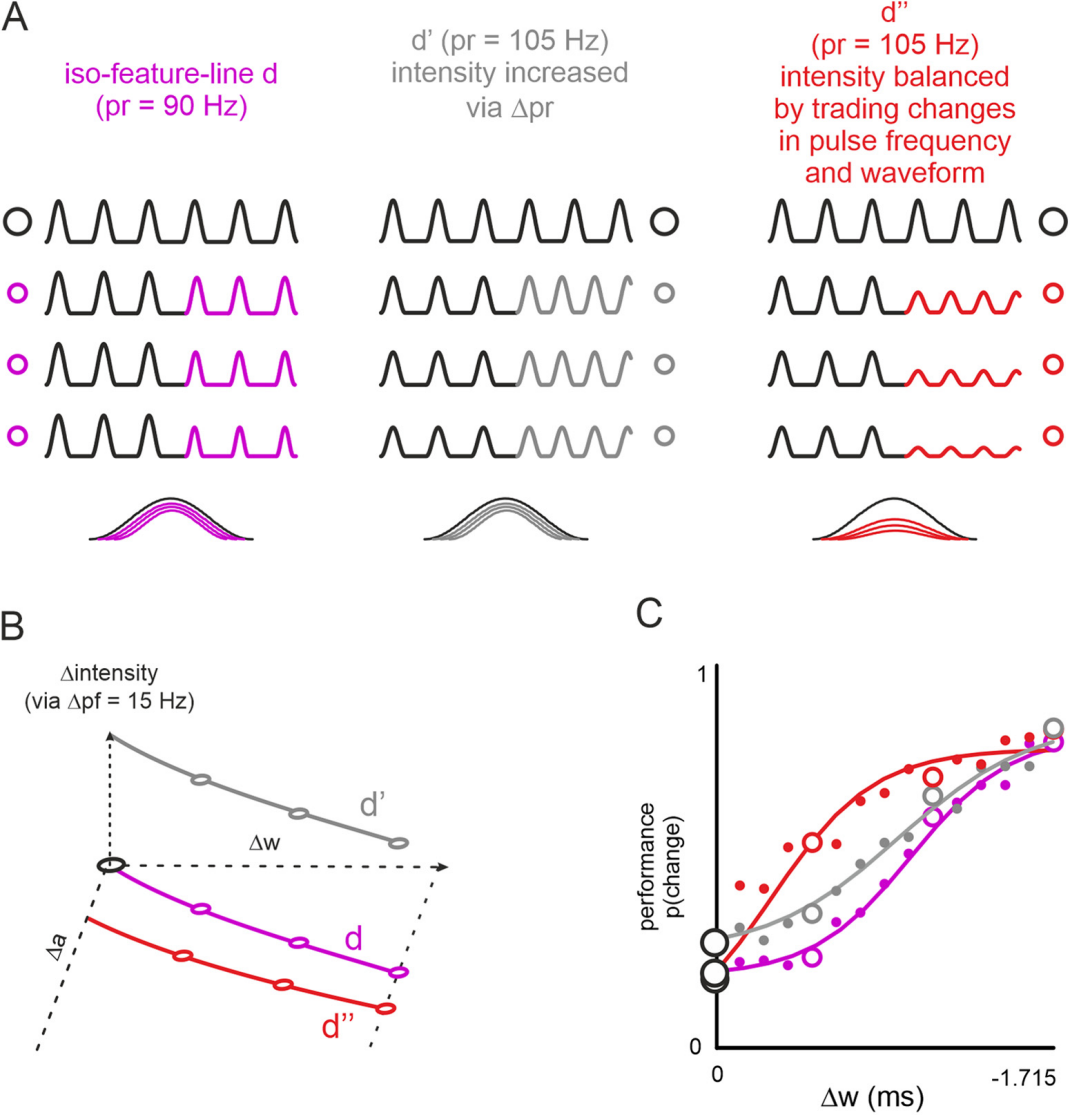
Local cues dominate global cues. Trading shape and rate changes within single trials. ***A***, Example stimuli from the three stimulus sets used in this experiment (only cut-outs around the stimulus change shown for clarity). Line d (magenta), Identical to Figure 2 (rate 90 Hz). Line d’ (gray), Pulse shape is identical to line d but rate of comparison stimulus was increased to 105 Hz. Line d’’ (red), Pulse rates as in line d’ (i.e., 105 Hz), but pulse amplitude was reduced to match the intensity of line d’’ to the reference. ***B***, The iso-feature-lines from which stimuli were picked. The circles refer to the example stimuli shown in panel ***A***. ***C***, Population performance and logistic fits on the three stimulus sets. The circles refer to the example stimuli shown in ***A*** (and also marked in ***B***).

**Figure 4.**
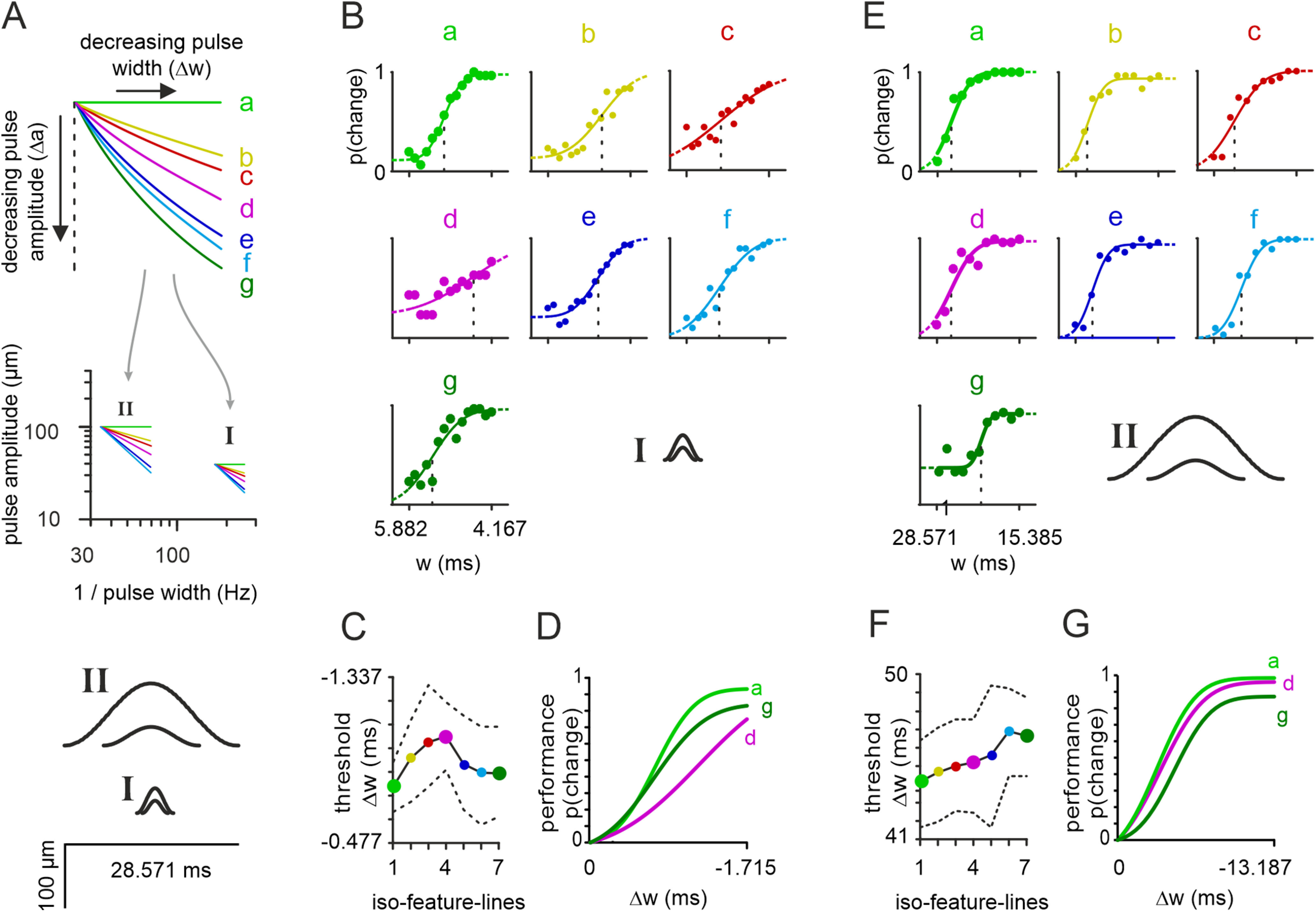
The tactile system does not use a unique preferred coding variable. ***A***, top, The stimulus space with iso-feature-lines placed in two different locations. Center, Range I was used to measure data presented in Figures 2, 3 and in panels ***B–D***. Its reference stimulus was a=40μm;w=5.882ms;pulserate=90Hz. Range II was used to measure data presented in panels ***E–G***. Its reference stimulus was a=100μm;w=28.571ms;pulserate=30Hz. Note that the iso-feature-lines in the center graph are the same as above but plotted on double log scale (they become straight lines by doing that). Bottom, the extreme pulse shapes of Ranges I and II are plotted on the same scale. The pulses found in the upper left and the lower right corner in each Range are shown. ***B***, Performance of a representative participant and logistic fits on all iso-feature-lines Range I. ***C***, Threshold of population performance (*n* = 10) on iso-feature-lines in the Range I. The colors refer to the iso-feature-lines. The dots represent the population mean derived using Bayesian hierarchical modeling (see Materials and Methods) along with dashed line corresponding Bayesian 95% to confidence interval. ***D***, Logistic fits of the three stimulus sets on lines a, b, g (corrected for false alarms; see Materials and Methods). ***E–G***, Like ***B–D*** but data from Range II. The extreme pulse shapes from Ranges I and II are re-plotted in ***B–D***, and ***E–G*** for reference.

A comparison of performance between pulse rate change and pulse shape change (iso-feature-line d) is shown in Figure 2. A comparison of performance between pulse shape change (iso-feature-line d) and pulse rate-and-shape change (iso-feature-lines d’, d’’) is shown in Figure 3. A comparison of performance between pulse shape change (all iso-feature-lines a–g) in stimulus Ranges I and II, is shown in Figure 4.

### Vibrotactile stimulation

We applied passive vibrotactile stimuli (i.e., no finger movement) to the distal pad of the left index finger, using a plastic circular disk of 2.9-mm diameter attached to a galvo-motor (Cambridge Technology; model 6220H). The galvo-motor was driven by a custom-made amplifier, which reproduced highly precise displacements. We calibrated the displacements of the galvo-motor using a laser distance estimator that is sensitive to displacements at the micron resolution. We used MATLAB to generate the stimulus waveform and control the galvo-motor movement by passing voltage waveforms through an analog output channel digitized at 40,000 samples/s at 12-bit resolution via a National Instruments PCI-MIO-16E-1 I/O board.

Pulsatile stimuli were constructed by using one period sinusoids (waveform of a sinusoid extracted from one of its minima to the next) as was done before (Gerdjikov et al., 2010). Two manipulations were done that involved a change in pulse width and/or amplitude. For pulse width, the sinusoids used were drawn from two different frequency ranges: in stimulus Range I, frequencies chosen were between 170 and 240 Hz in steps of 5 Hz, resulting in 15 pulse waveforms that varied in pulse width between 1 s/170 Hz = 5.882 ms and 1 s/240 Hz = 4.167 ms, and the pulse amplitude ranged between 20 and 40 μm, whereas in stimulus Range II, frequencies chosen were between 35 and 65 Hz in steps of 3 Hz, resulting in 10 pulse waveforms that varied in pulse width between 1 s/65 Hz = 15.385 ms and 1 s/35 Hz = 28.571 ms, and the pulse amplitude ranged between 30 and 100 μm. A third manipulation left pulse waveform untouched but changed the pulse frequency (rate), i.e., the inverse of interpulse intervals. Thus, we created three experimental conditions; namely, pulse shape condition (that involved manipulations 1 and 2, mentioned above), pulse rate condition (that involved manipulation 3), and pulse rate-and-shape condition (which is a combination of all the above manipulations; Table 1).

The stimulus for each trial was a seamless concatenation of two pulse trains both 500 ms in duration. The first train of pulses in all trials throughout this study was called the reference stimulus; the pulse rates of the reference stimulus in stimulus Ranges I and II were 90 and 30 Hz, respectively. The pulse shape of the reference stimulus had a width of 5.882 ms and an amplitude of 40 μm for stimulus Range I; in the case of stimulus Range II, the width and amplitude was 28.571 ms and 100 μm, respectively. The second train of pulses was either the same as the reference stimulus (which created the “no-change” trial type) or it involved one of the manipulations mentioned above, which are called the comparison stimuli and this created the “change” trial type (see Fig. 2*A*, the traces are to scale, but for purposes of visualization the traces contain only three pulses prechange and postchange).

Participants rested their arm on a platform which could be raised or lowered depending on their comfort. To prevent finger movements, the index finger was clamped in a finger housing using a cleft as a rest for the fingernail, in addition, a double-sided tape affixed the plane of the fingernail to the ceiling of the housing. Once the testing finger was securely positioned, we used a tri-axis micromanipulator to adjust and attach the galvo-motor to the distal pad of the left index finger such that the circular disk area was completely covered by the fingertip skin. During the experiment, only the testing region of the fingertip touched the circular disk, we ensured that no other part of the galvo-motor touched any part of the participants’ finger. We asked participants to trim the nail of their testing finger to prevent any possibility of their nail touching any part of the stimulator. The arm platform as well as the galvo-motor platform were separate from each other and seated on an anti-slip and anti-vibration mat. To achieve our definition of “1-mm depth of indentation,” first the experimenter moved the tri-axis micromanipulator closer to the participants’ fingertip until the participant reported feeling the contact of the stimulation disk, and then, using the micromanipulator, the disk attached to the galvo-motor was further indented by 1 mm. From there on, the stimulus pulses further indented the skin.

### Perceptual tasks and psychophysics procedure

Table 1 provides a quick glance at all the experimental conditions. The participants were instructed to indicate their decision in a yes/no fashion whether they perceived a change in the stimulus. In other words, if the comparison stimuli followed the reference, then it was a change trial, and “yes” would be the correct response; however, when the reference stimulus continued for the whole stimulus duration (i.e., instead of a comparison stimulus, the reference stimulus followed the reference again), it was a no-change trial, and “no” would be the correct response. Participants pressed one of two buttons (yes/no) on a wireless presenter clicker with their right hand. They received auditory feedback tones (high pitch for correct and low pitch for incorrect responses) after each trial, delivered through wireless headphones. All experiments were conducted in a block-wise fashion. Each experimental session contained three blocks, where each stimulus level was presented 10 times per block (i.e., 3 × 10 trials per stimulus level for the whole session, regardless of the experimental condition). There were equal number of no-change trials in total in every block, and the change as well as the no-change trials were presented in a pseudo-random order. Apart from the auditory feedback tones, participants also saw their performance as the total percent correct for that block. The intertrial interval started after the participant’s response and lasted for 5 s. After each block participants had to take a minimum of 2-min break during which they were encouraged to stand up and walk around.

Participants were tested in 3 different experimental conditions – pulse shape change (in two stimulus ranges), pulse rate change, and pulse rate-and-shape change (Table 1). In the pulse shape change experiment, we tested each participant on stimulus changes drawn from seven iso-feature-lines shown in Figure 2*B* (seven sessions were performed, testing one iso-feature-line per session; see Table 1). Each session, in the stimulus Range I, contained 420 change and 420 no-change trials, and in the stimulus Range II, there were 300 each of change and no-change trials. The iso-feature-lines contained stimuli, from which we extracted three local and six global encoding variables. The three local variables were (maximum position, maximum velocity and maximum acceleration). In the stimulus space depicted in Figure 2*B*, the three local encoding variables were identical to the one obtained from the reference stimulus on iso-feature-lines a, d, and g, respectively. The six global encoding variables quantified intensity, e.g., the mean of the vibrotactile signal; they were calculated always across the entire 500-ms stimulus. The following intensity formulations were tested (in brackets we detail the respective iso-feature-line in the stimulus crossing space; Fig. 2*B*): mean absolute velocity (a); mean squared velocity (b); mean absolute velocity taken to the power of three (c); mean absolute acceleration (d); mean squared acceleration (e); mean absolute velocity taken to the power of 3 (f). In two instances an iso-feature-line of a local encoding variable was found to be congruent with one of the global encoding variables (“maximum position” = “mean absolute velocity”; “maximum velocity” = “mean absolute acceleration”); hence, only seven iso-feature-lines were tested in total. In the pulse rate change experiment, stimulus changes were exclusively based on changes in pulse frequency ( Δpf; Fig. 2*A*). There were nine change stimulus levels and one no-change stimulus, yielding 540 (i.e., 270 change and equal number no-change) trials per session (30 trials per stimulus). Finally, in the pulse rate and shape change experiment, we tested participants in two variations of iso-feature-line d, which we tag as iso-feature-line d’ and d’’. In both variations, there was an additional change (along with the change in pulse shape) in pulse frequency in the comparison stimuli; the pulse rate of comparison stimuli was 105 Hz (i.e., 15 Hz higher than the reference pulse rate of 90 Hz), a value that was far subthreshold for all participants (see Results). The aim of this experimental condition was to disentangle effects on performance of two identical intensity changes, one time realized by increasing pulse rate (manipulation of the global variable; iso-feature-line d’ in Fig. 3*B*), the other time by altering pulse shape (manipulation of the local variable; iso-feature-line d’’ in Fig. 3*B*). In the pulse rate and shape change experiments, the number of trials were identical to the one used in the pulse shape change experiment.

Participants wore padded headphones throughout the study to reduce ambient acoustic noise and receive auditory feedback. In experiments that involved a change in pulse rate, there was a very low acoustic signal emitted by the galvo motor reflecting the change in pulse rate, hearable when locating the unpadded ear next to the motor. To securely rule out the possibility of unwanted auditory cues as experimental confound, we played out loud white noise from a speaker next to the galvo in the two experiments containing pulse rate changes. Participants were uninformed about the details of stimulus change. Following the completion of pulse rate and shape change experiment, when asked, none of the participants was aware that the pulse rate (along with the pulse width and amplitude) of the target stimuli changed.

### Statistical analysis

To each participant’s performance (proportion reported “change”) in each task, using the dedicated analysis software *psignifit* (Wichmann and Hill, 2001), we fitted a mixture model cumulative normal psychometric function *P*(x) of the following form:

P(x)=γ + (1−δ−γ)p(x),

where *p*(x) is the sigmoid modelled as cumulative Gaussian – which includes the threshold (i.e., the mean) and width (i.e., the SD) parameters, γ parameter is false alarm (lower asymptote), and δ parameter is the lapse rate (upper asymptote). The *psignifit* algorithm computed the maximum likelihood of each psychometric function generated from the combination of each of the above-mentioned parameters. By marginalizing over the γ, δ, and width parameters *psignifit* yielded a posterior probability density function (PDF) over the threshold parameter θ, i.e., where the psychometric functioned crossed 50% correct. For each participant’s point estimate of their performance on each iso-feature-line, we chose the stimulus value corresponding to the mode of the threshold PDF. For illustration purposes as well as graphical comparison, we performed false alarm correction (see Klein, 2001).

Next, to estimate the population mean for each iso-feature-line, we implemented a Bayesian Hierarchical analysis (see Tong et al., 2016). We represented the participants’ threshold as normally distributed over the whole stimulus range (pulse width from 5.882 ms down to 4.167 ms) with unknown mean μ and SD σ (ranging between 0.5 and 30). The probability of a participant’s data given (μ, σ) can be written as:

$$p\left(d_{i}\text{|}\mu ,\sigma \right)=\overset{}{\underset{\begin{array}{c} i=stimuli\\ 5.882-4.167ms \end{array}}{\int }}p\left(d_{i}\text{|}\theta \right)p(\theta |\mu ,\sigma )d\theta .$$

Here, the first term in the integrand is proportional to the threshold PDF of each participant (acquired from *psignifit*), because we assume a uniform prior over all possible values of θ (i.e., 5.882–4.167 ms). Thus, the likelihood of joint distribution of each (μ, σ) of complete dataset *D*, including all 10 participants can be represented as:

$$p\left(D\text{|}\mu ,\sigma \right)=\overset{10}{\underset{i=1}{\prod }}p(d_{i}|\mu ,\sigma ).$$

Finally, we marginalized over σ and obtained a PDF of the population mean; we report the mode of this distribution as the mean of the population along with the Bayesian 95% confidence interval.

### Simulation of primary afferents

We used TouchSim (v1.1; available at https://github.com/bensmaiaLab/touchSim), a freely available simulation tool based on indentation biomechanics followed by integrate and fire neurons (Saal et al., 2017). The algorithm allows to selectively model the primary afferents in the glabrous skin across the entire index finger with realistic densities of end organs of the three primary afferent classes slowly adapting type 1 (SA1), rapidly adapting type 1 (RA1), and rapidly adapting of the Pacinian corpuscle (PC)-associated type (also called rapidly adapting type 2). The model accepts skin indentations of adjustable size and at location on the glabrous skin of the finger. Our simulations used the approximate location ([−10,1], in coordinates of the algorithm) and extent of the indenting disk (diameter of 2.9 mm). The stimulation disk was placed at a preindentation depth of 1 mm below the skin surface. Stimuli presented were identical to those applied to the humans’ fingertip. The model ran simulations of 564 SA1 (Merkel cells), 933 RA1 (Meissner Corpuscles), and 192 PCs, distributed across the fingertip.

**Figure 5.**
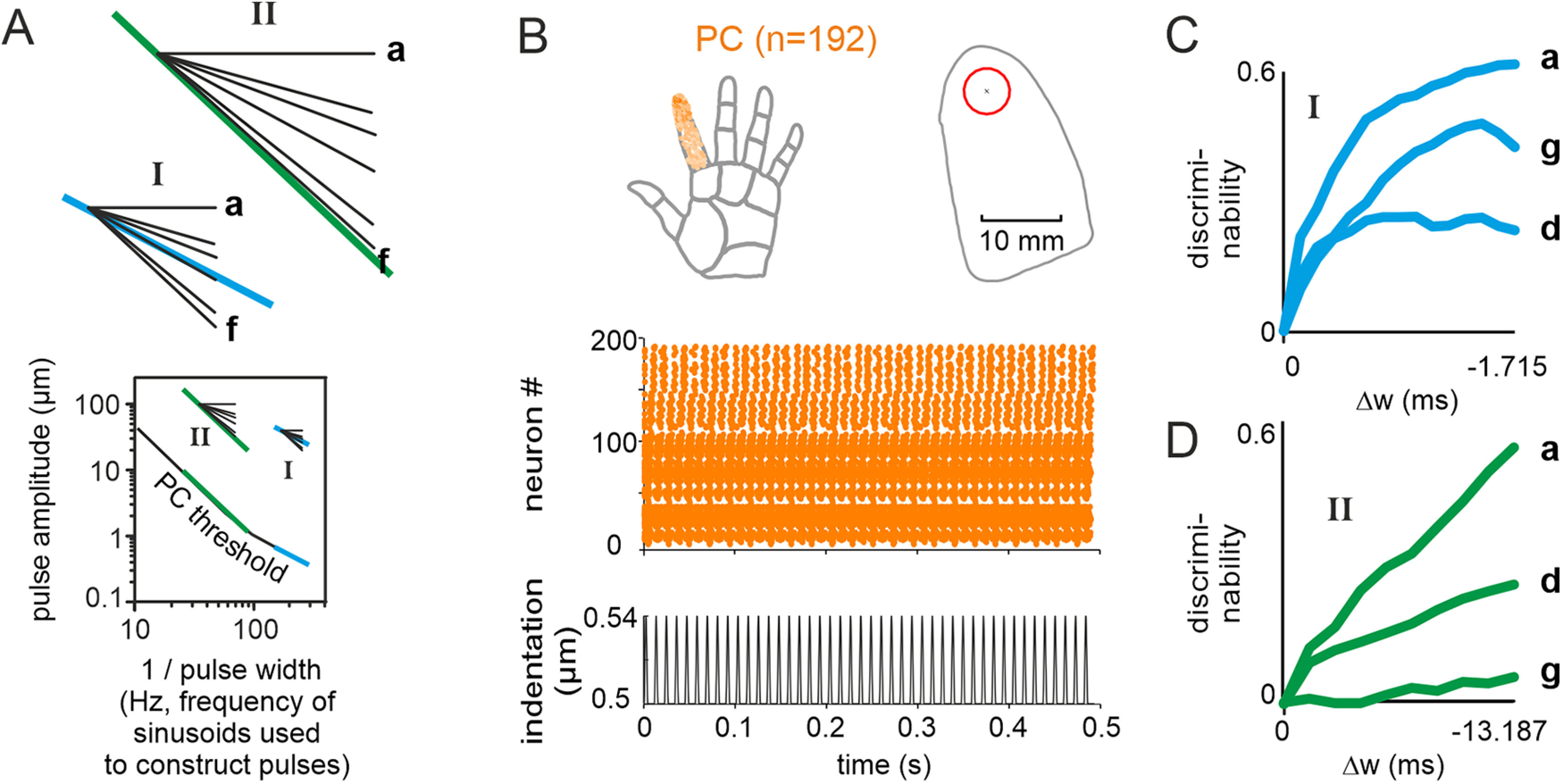
Neurometrics based on simulated population PC activity recreates relative performance on iso-feature-lines. ***A***, Schematics showing the location of the stimulus ranges used here with respect to the threshold curves for PC spiking obtained by Freeman and Johnson (1982; threshold is defined as the stimulus that evokes the first spike). Iso-feature-lines from pulsatile stimuli and the sinusoids used in the cited study can be plotted together in one graph, because (1) pulses are sinusoidal; and (2) PC afferents do not show firing rate adaptation across pulses. The iso-response lines of PCs (green and blue lines) are approximated by constructing parallel lines traversing the stimulus Ranges I and II. The plots above the graph are the Ranges I and II with iso-feature-lines for intensities a-f and approximated PC-iso-response lines magnified. Collinearity exists roughly with line d in Range I and line f/g in Range II (aligned to worst performances observed in these ranges; compare Fig. 4*B-D* and *E–G*, respectively). ***B***, Model parameters and output of PC population (TouchSim, version 1.1; Saal et al., 2017). Center, Raster plot of one stimulus presentation (*n* = 192 simulated PCs). Top left, Location of afferents simulated across the entire index finger [color indicates the distribution of relative activity (dark color = high evoked activity; pale color = low evoked activity)]. Bottom, An example pulsatile indentation stimulus, identical to the one applied in the psychophysical experiments. Top right, As in the human experiments, stimuli were delivered via a circular disk of 2.9-mm diameter (red; gray outline = distal segment of the index finger). The location of the disk in units of the model was [−10,1]. ***C***, Simulated neurometric curves (scaled as discriminability; see Materials and Methods) using stimuli on lines a, d, g Range I. ***D***, Same but for simulations based on Range II. Spike count responses of primary afferent models composed of SA1, RA1, and PC primary afferents are shown in Extended Data Figure 5-1.

**Figure 6.**
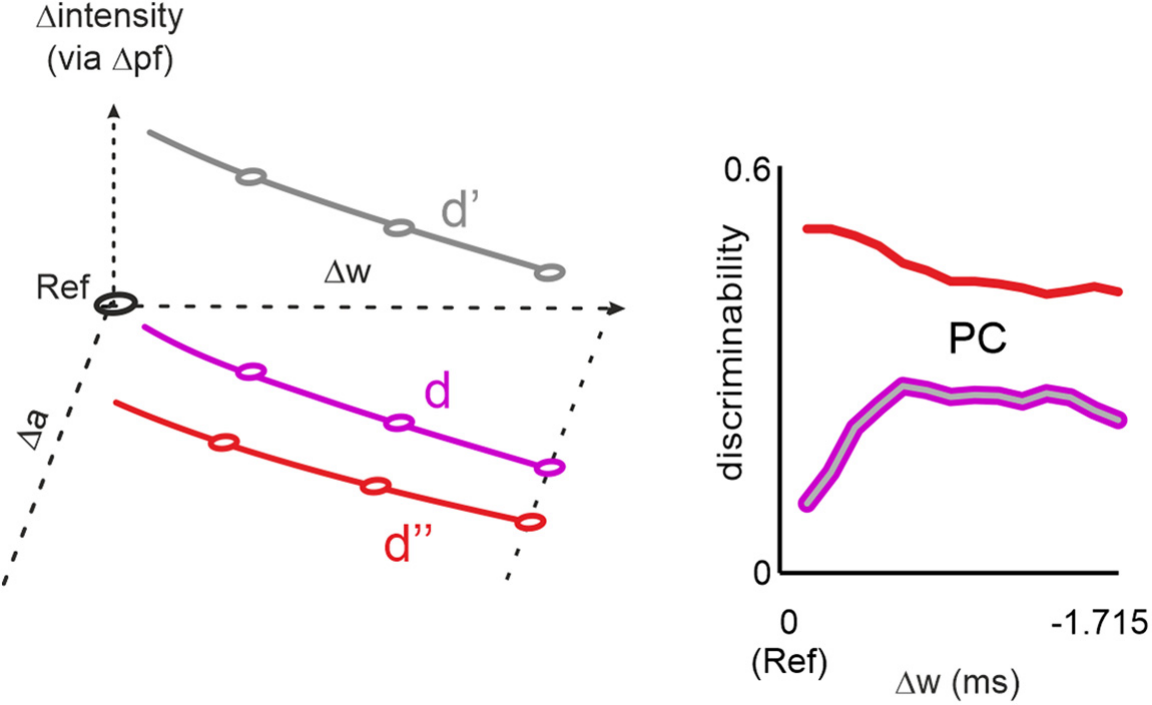
Neurometrics based on simulated population PC activity recreates relative performance on stimulus sets mixing rate and shape changes (see Fig. 3 for human performance). Left, Stimulus space replotted from Figure 3*B*. Lines d, d’’ are located in the plane spanned by Δw and Δa. Line d’ is equidistant along the vertical dimension to lines d, d’’ [i.e., in terms of intensity, the change from line d to d’ (realized by additional pulses), and the change from line d’ to d’’ (realized by shape changes), is fully balanced]. Right, Simulated population neurometric performance (*n* = 192 PCs; compare Fig. 5*B*). The congruence of the responses to stimuli picked from d, d’ (magenta and gray) is trivially expected, as the responses were calculated from modelled PC responses to single pulses (summing spikes across pulses would invariably lead to error-free performance, i.e., discriminability = 1, which is non-compatible with psychophysical results). The increase of discriminability when compensating intensity increment because of added pulses by reducing pulse shape, qualitatively reflects human psychophysical performance (compare Fig. 3). Ref = reference, i.e., no-change stimulus.

The model returned spike trains from each of the simulated primary afferents, which we analyzed further. We modelled responses for all stimuli (reference and comparison) used in this study (Figs. 5, 6; Extended Data Fig. 5-1). All analyses presented are based on averages of 100 model runs (= stimulus presentations). The first coding symbol extracted was either the “global” spike count, i.e., the sum of spikes evoked across the stimulus length (500 ms) of reference and comparison stimulus. This gave an ensemble of 100 spike responses (one per run/trial). The second coding ensemble read-out was composed of “local” spike responses to each pulse [this gave an ensemble of 45 × 100 responses (number of pulses times trials)]. Neurometric performance was estimated by calculating the area under the curve (AUC) of the receiver operating characteristic (Stüttgen et al., 2011) using the response distributions obtained from the model runs with reference and comparison stimulus. AUC is the probability of a correct stimulus identification of a binary classifier confronted with a random pick of spike responses from the total sample. In the current framework, where only a change of stimulus had to be detected AUC values of 1 and 0 are equivalent and indicate optimal performance, while a value of 0.5 indicates random performance. We therefore calculated discriminability d [ranging from 0 (random) to 1 (100% discrimination)] as d=2*|AUC–0.5|.

10.1523/ENEURO.0263-21.2021.f5-1 Extended Data Figure 5-1 Population spike responses of all three modelled primary afferent classes. ***A***, The model contained 564 slowly adapting primary afferents type 1 (SA1, green), and 933 rapidly adapting primary afferents of type 1 (RA1, blue), all located close to the indented site on the fingertip (conventions of the hand drawing as in Fig. 5, for RA1 only the distal phalanx of the index finger is shown). Spiking in response to one trial (reference stimulus of Range I, duration 500 ms) is presented as raster plots. Each line represents the spiking of one modelled neuron; note that a majority of neurons did not spike at all to this stimulus (for PC data see Fig. 5). ***B***, Summary response profiles for all three modelled primary afferent populations (rows of graphs). Each graph plots on the ordinate either the spike numbers (top, mean ± SD) or the mean absolute difference spike numbers between reference and comparison stimulus (bottom), summed across the neuronal population. The abscissae hold the reference stimulus (leftmost data point) as well as all comparison stimuli (to the right with decreasing pulse width). Red color labels data from Range I, green indicates data from Range II. Each column of graphs presents the responses to one iso-feature-line (labeled as in Fig. 2). The iso-feature-lines yielding worst population performances of participants for the two ranges (d, g) are marked at the bottom (compare Fig. 4). The ovals mark the indiscriminate responses of the PC population across reference and comparison stimuli to these iso-feature-lines. SA1 and RA1 afferents show indiscriminate responses to stimuli on line a (SA1) and b (RA1), and thus, by themselves, are unable to explain human performance. Download Figure 5-1, TIF file.

Primary afferents are known to lack spike adaptation (Bensmaïa et al., 2005; Leung et al., 2005). Therefore, local and global coding symbols gave near identical results as long as the pulse frequency was not varied. As soon as differences in pulse rates were applied, however (data presented in Fig. 6), the global coding symbol (sum of spikes across 500 ms) of all classes of primary afferents would indicate all changes in near error-less fashion (i.e., discrimination close to 1). This has been noted before (Stüttgen et al., 2006; Gerdjikov et al., 2010; Arabzadeh et al., 2014), and excludes that the perceptual system is able to use the full global information present on the level of primary afferents. Figure 6, therefore, reports exclusively results obtained with calculating the ensemble generated by response to single pulses.

## Results

We used a yes/no psychophysical change detection task to test whether humans can discriminate pulsatile skin indentations. The participants were presented with a 1-s-long pulsatile stimulus and were asked whether they felt a change in the middle of this vibration. The stimuli were composed of two 500-ms-long pulse trains that were seamlessly concatenated, a “reference” followed by a “comparison” stimulus (correct response “yes”), or the “reference” again (correct response “no”). Stimuli were delivered orthogonal to (i.e., indenting into) the skin of the left index fingertip. The comparison stimuli changed from the reference in three different ways– a change in pulse rate, shape, or rate-and-shape (Fig. 1*B*; for an overview about all experimental variables used in this study, see Table 1).

### Stimulus space, iso-feature-lines, and terminology

The first variable characterizing a change between reference and comparison stimulus, pulse rate, determines the number of pulses per time interval (Fig. 2*A*, blue), and can only be decoded by integrating across pulses. It, therefore, requires “temporally global” decoding, either summing signal (intensity) or frequency contributions (spectrum). The second variable was the shape of the individual pulses, which came in two variants, namely changes in pulse amplitude and/or width (Fig. 2*A*, magenta). It is noteworthy that a seemingly local manipulation, in principle detectable instantaneously by observing pulse kinematics, also affects the global variable, the intensity (i.e., the signal summed across pulses; Fig. 1*B*). Individual pulses always had the sinusoidal shape (single-period sine-wave starting from one minimum and ending at the next), starting from the resting position (at 1 mm skin indentation), and protruding further toward larger depths. For pulse shape, two parameters were manipulated independently, pulse width and amplitude, by changing the frequency and/or amplitude of the sinusoid that was used for constructing the pulse ( pulsewidth=1/frequency). To disambiguate our terms, we use “pulse rate” to refer to the number of pulses per time unit exclusively, and we reserve the terms “pulse shape” (i.e., “amplitude” and “width”) as a proxy for the amplitude and frequency of the sinusoid used to generate the individual pulses. Whenever we use the term “frequency,” we refer to long sinusoidal stimulation used in classical studies or to the frequency of the sinusoid used to construct our pulses. Our approach implies that pulse rate and pulse shape are independent experimental variables as long as pulse rate is less than the frequency of the sinusoids used to construct the pulses; this approach is different from traditional frequency discrimination experiments using long sinusoids, where changing the pulse rate inadvertently changes the pulse shape.

Pulse amplitudes and widths were chosen from a two-dimensional stimulus space shown in Figure 2*B* (pulse rate: 90Hz; pulse width: w∈[4.167,5.882]ms; pulse amplitude a∈[20,40]μm). The reference stimulus was composed of the widest and highest pulses ( w=5.882ms;a=40μm), located at the upper left corner of the stimulus space (large circle), whereas the comparison stimuli were composed of pulses that were reduced in width ( w) and/or amplitude ( a). In preliminary experiments (using lab members as participants; data not shown), we found that our stimulus space roughly divides into a region with relatively poor and graded responses (orange), and another with extremely good performance (gray). Whereas the gray zones predicted ceiling performances in participants and would be uninformative experimentally, the responses within the orange field, which is sensitive to shape parameters, promised to unearth evidence toward principles of pulse shape coding. The performance on stimuli bordering the gray high-performance fields was still very good and it decayed toward a line of “worst” performance in the center of the orange field (Fig. 2*B*).

The orange stimulus space covers a wide range of pulse width and amplitude combinations, from the upper left corner (our reference stimulus) where the pulses are wider and higher compared with the ones down in the lower right corner where the pulses are lower and narrower (i.e., steeply rising pulses). That is, the orange stimulus space is characterized by stimuli that trade decreasing amplitude with increasing steepness. In fact, starting from the reference stimulus in the upper left corner, it is possible to trace out lines through the orange stimulus space, on which important coding variables (“features”) remain unchanged, we tag them as “iso-feature-lines.” In this study, we considered seven such iso-feature-lines (labeled a–g in Fig. 2*B*). Amongst them are six iso-feature-lines (a to f) that hold variants of global intensity variables constant, defined as “mean velocity” (line a), “mean squared velocity” (line b), “mean cubic velocity” (line c), “mean acceleration” (line d), “mean squared acceleration” (line e), and “mean cubic acceleration” (line f). Note that to increase clarity, we omitted the attribute “absolute” (i.e., directions and signs of velocity and acceleration are not considered – all variables are positive valued). Moreover, the seven iso-feature-lines also comprise three lines that hold local variables constant describing the “maximal position” (i.e., maximal displacement), “maximal velocity,” and “maximal acceleration” of a pulse (iso-feature-lines a, d, and g, respectively). The first two respective local iso-feature-lines are in fact congruent with the global iso-feature-lines mean velocity and mean acceleration (lines a and d), whereas “maximal acceleration,” the third local variable, is characterized by the bottom-most iso-feature-line (g) in Figure 2*B*. Note that throughout this article we use the letters a–g to label iso-feature-lines, but also to refer to the intensities and kinematic variables held constant on them.

Having identified the relevant stimulus range we decided to pick comparison stimuli located on the seven iso-feature-lines. We reasoned that if one of these encoding variables a critical for performing the change detection task, the performance on the corresponding iso-feature-line should be poor compared with the other ones. To quantify this, we designed our testing sessions under the presumption that decreasing pulse width (from the pulse width of the reference stimuli) will increase the participants’ probability of detecting a change and the pulse width at which the probability to detect the change is 50% will be the threshold. In other words, in all the iso-feature-line sessions we reduced the pulse width of the comparison stimuli (while adjusting for the pulse amplitude as guided by the iso-feature-line definition) to finally identify the iso-feature-line session that yielded the highest threshold. We tested each participant through seven sessions corresponding to each iso-feature-line; the sessions were randomized such that each participant was tested on a unique sequence of sessions probing the seven iso-feature-lines (Table 1).

### Comparison between isolated changes in pulse rate and shape

In anticipation of results that we will report in detail further below, we found that among the sessions on the seven iso-feature-lines, stimuli on line d gave rise to the highest threshold, i.e., worst performance. To start comparing the effect of local and global cues on performance, we selected stimuli on line d, with the worst performance among the stimulus sets containing local cues (Fig. 1*A*, orange; compare Table 1 under “shape change condition”), and pitted them against stimuli that are devoid of local cues, i.e., reference and comparison stimuli containing pulses of identical shape but different rate (Fig. 1*A*, blue; compare Table 1 under “rate change condition”). In the blue stimulus set the pulse rates changed from 90 Hz (reference) to 135 Hz in 10 stimulus steps (i.e., 90, 95, …, 130, 135 Hz). We present data obtained from eight of nine people tested, as the ninth participant did not give us any logistic fit that crossed the probability of 0.5 of detected change. On average, participants required a pulse rate change of 35% (SD 13%) to reliably detect it 50% of the time. The orange stimulus set included 15 pulse shapes, whose width varied from 5.882 to 4.167 ms (the interpulse intervals of reference and the comparison stimuli were identical), and amplitudes were defined by line d (Fig. 2*A*, right, and panel *B*). For detection of changes in the pulse shape task, we obtained valid psychometric curves (threshold higher than detection probability of 0.5) from all 10 participants.

Although one set of experiments dealt with changes in pulse rate and the other with pulse shapes, the performance on the two types of stimulus changes can be plotted in one graph according to their intensity content. Figure 2*C* presents the mean performance of the population of participants in the two experiments plotted across intensity formulated as mean velocity. The success of 8 out of 9 participants to detect pulse rate changes first of all demonstrated that humans in principle are able to use global variables as a basis for their decision. However, the comparison of the two behavioral datasets based on change of intensity in Figure 2*C* suggested that pulse shape changes (magenta) were detected in a different way (i.e., had a lower intensity threshold) than the ones based on pulse rate (blue).

However, the premises for the comparison made in Figure 2*C* are, first, that intensity is actually a coding variable, and second, that its formulation as mean velocity is correct. The first premise is firmly supported by a host of studies starting from early times of tactile research (LaMotte and Mountcastle, 1975). The second premise is definitely weaker, but has been supported by work comparing some of the possible formulations (Arabzadeh et al., 2003; Hipp et al., 2006; Gerdjikov et al., 2010). Nevertheless, in many studies on the tactile system the formulation of intensity was chosen for reasons of parsimony or tradition. In order to strengthen our argument, we took the opportunity that was provided by the outline of our relevant stimulus range and the set of iso-feature-lines running through it (Fig. 2*B*), and performed a more systematic analysis: we reasoned that the true intensity formulation for indentation stimuli (if it exists) must be associated within this stimulus range, where relevant intensity formulations do not vary considerably and performance accordingly is relatively poor. We therefore repeated the plot of Figure 2*C* with combinations of data from all seven iso-feature-lines (colored curves in Fig. 2*D*), and scaling them to the six intensity formulations a–f, whose iso-feature-lines span the whole stimulus range (the six graphs in Fig. 2*D*). We did not find any intensity formulation that would group psychometric curves consistently close to the one from pulse rate changes. They are either concentrated at sites indicating far better performance than rate changes (e.g., mean velocity and mean acceleration, labeled a and d), or they are dispersed across a large intensity range (e.g., “mean squared” or “cubic acceleration,” intensities e and f). We argue that a consistent grouping of curves around the one found with rate changes would be required to conclude that the respective intensity formulation was perceptually dominant. The absence of such a grouping suggests that none of the intensity variables singularly determined the performance on the present tasks. The most conspicuous mismatch was the one found when scaling to intensity d (mean acceleration; Fig. 2*D*, lower left). Actually, the poor performance on line d, mentioned before, had made mean acceleration a major candidate for intensity-based coding. However, the huge difference in performance between rate and shape change stimuli, when scaling with this variable, casted serious doubts on its relevance. From these initial results we, thus, suggest that the observed performance differences are likely based on differences in local pulse shapes rather than intensity.

### Comparison of changes when presenting simultaneous changes in pulse rate and shape

So far, we tested shape and rate changes in different sessions, leaving room for the possibility that the participants (or their tactile systems, respectively), were able to adapt to these different tasks. We therefore aimed to support the finding in a more direct way, trading pulse rate against shape within the same comparison stimulus (Fig. 3*A*). As a starting point for this experiment, we chose again iso-feature-line d (Fig. 3*B*). The poor performance on this line would predict that the variables kept constant on it (i.e., global mean acceleration or local “maximal velocity”) are likely relevant for coding. Further, the poor performance would provide added pulses a good chance to result in measurable improvements in performance (conversely, basing the experiment on iso-feature-lines with stronger performance would run the risk that performance there is already saturated preventing the assessment of improvement by adding pulses). Adding pulses of identical shape to comparison stimuli on line d generated line d’ (Fig. 3, gray; also see Table 1, pulse rate-and-shape change experiment), which keeps being a local iso-feature-line identical to line d (because of identical pulses), but introduces differences in global intensity (via the increased pulse rate). We selected an increase of pulse rate of 15 Hz (i.e., comparison stimuli on line d’ have a pulse rate of 105 Hz as opposed to 90 Hz for line d), which is subthreshold based on participants performance in the pulse rate change experiment (compare Fig. 2*C*, the stimulus is indicated by the leftmost blue hexagon). Expectedly then, increasing pulse rate did not significantly improve the performance in iso-feature-line d’ over what was seen in iso-feature-line d [change of threshold from 1.272 ms (SD 0.27 ms) to 1.101 ms (SD 0.19 ms); *t* test: *t* = −2.084, *n* = 8, *p* = 0.08; effect size: discriminability = 0.74]. In other words, the participants largely failed to use the increased global intensity variable in the comparison stimuli and continued informing their perceptual decisions using local cues. Therefore, our next manipulation aimed at deciding whether the same intensity change, this time realized by reducing pulse shapes further (thereby, strengthening local cues), would have a better effect on performance. To test this, we compensated intensity (that was previously increased via rate change in line d’) by reducing the pulse shape. This resulted in line d’’ (Fig. 3, red; also see Table 1). Performance on line d’’ significantly improved in comparison to line d’ [gray to red; change of threshold from 1.101 ms (SD 0.194 ms) to 0.764 ms (SD = 0.368 ms); *t* test: *t* = 2.868, *n* = 8, *p* = 0.024; effect size: discriminability = 0.98]. That is, relative to the reference (d, magenta), increasing the pulse rate had no effect on performance (d’, gray) whereas changing the pulse shape enhanced it (d’’, red). In other words, there is no significant difference in performance between d’ and d, likely because there is no difference in the local cue. In contrast, there is a significant difference in performance between d’ and d’’ likely because here local cues vary, while intensities are kept the same, and rate change alone has been shown before to be perceptually ineffective.

### The tactile system does not prefer a specific kinematic variable

Translating the width of our sinusoidal pulses into frequency, i.e., the variable classically considered when testing the tactile system with long sinusoidal stimuli, it becomes evident that so far we only probed a small portion of the wide tactile range accessible to the human tactile perceptual system (Bolanowski et al., 1988; for a justification of the comparison of stimulus ranges composed of sinusoids in the classical studies and our sinusoidal pulses, see Discussion). The subrange investigated thus far (Range I in Fig. 4*A*; pulse widths were between 5.882 and 4.167 ms) corresponds to sinusoidal frequencies between 170 and 240 Hz, which is well within the range of frequencies classically called “the Pacinian range”; a range that is dominated by responses of rapidly adapting primary afferents type 2, which innervate the PCs. We therefore aimed, firstly, to quantify performance on all iso-feature-lines of Range I, and secondly, compare it to a second range, the so-called “flutter range”, located at much lower frequencies, known to predominantly activate rapidly adapting primary afferents type 1 (Range II in Fig. 4*A*; amplitudes 100–30 μm and widths 28.571−15.385 ms⇔35−65Hz; see also Table 1). To provide an intuition about pulse shapes used in Ranges I and II, we plot the largest and the smallest pulses, defined by the top left (i.e., a reference stimulus pulse) and bottom right corners of the two ranges (Fig. 4*A*, bottom).

The performances of exemplary participants on the stimulus Ranges I and II (Fig. 4*B*,*E*), already hint that the preference does not generalize across the entire stimulus space. As mentioned before, iso-feature-line d (maximum velocity) yielded the worst performance in Range I (Fig. 4*B*), while in Range II the worst performance was seen with iso-feature-line g (maximum acceleration; Fig. 4*E*). Population data, thresholds as well as psychometric curves, confirm this impression (Fig. 4*C*,*D*,*F*,*G*). For statistical appraisal we computed the discriminability (ranging from 0 to 1; see Materials and Methods), as well as the *p* values obtained from a *t* test (two-tailed, paired samples), comparing thresholds from iso-feature-line d to all others for the Range I (discriminability = [0.96, 0.64, 0.06, ∼, 0.66, 0.82, 0.80]; order: line [a, b, c, d, e, f, g], note the fourth position is marked with “∼” as it is the comparison of line d with itself). The corresponding *p* values were *p* = [0.015, 0.044, 0.624, ∼, 0.357, 0.049, 0.055]; and number of tested participants were *n* = [10, 9, 9, ∼, 9, 10, 10]. Similarly for Range II, we computed discriminability comparing the iso-feature-line g (maximum acceleration) to all the other iso-feature-lines, and ordered the results as above: discriminability = [0.88, 0.78, 0.68, 0.64, 0.38, 0.14, ∼]; the corresponding *p* value in the *t* tests were p = [0.027, 0.086, 0.134, 0.166, 0.413, 0.989, ∼], and the number of participants were *n* = [10, 10, 10, 10, 10, 10, ∼]. The best fit logistic functions corrected for false alarm rates to the pooled data from 10 participants for the experiments on isolines (a, d, g) are shown in Figure 4*D*,*G*. The different sequence in performance can be readily observed from them (a→g→d in Fig. 4*D* vs a→d→g Fig. 4*G*). We note that both tasks were challenging for the participants. This fact was reflected by a consistently high false alarm rate of [0.12, 0.18, 0.16, 0.14, 0.09, 0.04, 0.07] in stimulus Range I, and [0.03, 0.03, 0.04, 0.04, 0.02, 0.04, 0.05] in Range II (same order as before). In summary, for human vibrotactile perception, the relative performance across iso-feature-lines was distinct in the two studied stimulus ranges, a result that does not argue in favor of a single consistent weighing of kinematic parameters for encoding; instead, participants seemed to switch between encoded variables to perform the task in the different stimulus subspaces.

### How is change detection related to primary afferent activity?

The schematic in Figure 5*A* plots again our stimulus subspaces within the larger perceptual range, but now adds the threshold curve of PC primary afferents (Freeman and Johnson, 1982). As our stimulus spaces are quite small, relative to the curvature of the threshold curve, it is possible to approximate the curve with straight line segments, and thus, we arrive at “iso-response-lines” (Fig. 5*A*, green and blue lines). The primary afferent response to a pair of discriminanda located on an iso-feature-line is then governed by the angle with which an iso-feature-line runs relative to the afferent’s iso-response orientation. For example, a pair of stimuli located on an iso-feature-line would engage a class of afferents similarly if the iso-feature-line is parallel to the iso-response orientation, whereas the stimuli would engage the afferents most differently, if the iso-feature-line and iso-response orientation are orthogonal. The schematic comparison of the cited classical work to our data in Figure 5*A* demonstrates how the orientation of the PC threshold line can be transferred to our stimulus ranges and respective iso-feature-lines. It turns out that, to a first approximation, the angle between iso-response-lines and iso-feature-lines is different in the two stimulus subspaces (Fig. 5*A*, blue vs green lines). From here it is straight forward to hypothesize that minute changes in the angle of iso-response-lines and iso-feature-lines are responsible for the observed difference in preferred coding variables in Subspaces I and II (Fig. 4).

We put this hypothesis to the test by taking advantage of an established model of primary afferent responses based on quasi-static and dynamic skin mechanics linked to an integrate-and-fire neuronal model (Saal et al., 2017). The model very precisely recreates the classical threshold and entrainment curves of the three most important classes of primary afferents in primates (Saal et al., 2017; see their Fig. 7), and its output therefore, as argued above, can be compared with our human behavioral data. Importantly, the model accepts any waveform of indentation stimuli, it is sensitive to stimulation location, and it realistically simulates multiple end organs distributed across the glabrous skin. We only quantified the discriminability carried by spikes of the PC class of primary afferents (also called rapidly adapting type 2), as they qualitatively reproduced the main aspects of our behavioral results (Fig. 5), while the other two (SA1 and RA1) did not. (The spike responses of all modelled primary afferent classes are presented in the Extended Data Fig. 5-1.) We modelled the spike output of 192 PC afferents to our pulsatile stimuli, distributed in spatially realistic fashion across the index finger as shown in Figure 5*B*. We ran the model presenting the reference stimuli and all stimuli on the seven iso-feature-lines, covering both stimulus ranges presented to the human participants in this study. We plot the discriminability of population PC spike counts calculated from the number of spikes evoked by the reference stimulus versus that obtained with the comparison stimuli for three isolines in each stimulus range. We performed this analysis by summing across responses to all pulses contained in one stimulus (reference vs all comparison stimuli), to be consistent with the psychophysical experiments. However, because of the known lack of spike rate adaptation in primary afferents (for details, see Discussion), captured by the model, we did not find relevant differences when presenting single sinusoidal pulses or 500-ms-long pulsatile sequences to the model (the differences were smaller than the line thickness of the plots in Fig. 5*C*,*D*). So, for discrimination analysis, the quantification of primary afferent activity based on responses to single and repetitive pulses can be used interchangeably. Couching these long-known facts in the terminology introduced in this study, we can call primary afferents as “temporally local encoders.” The neurometric curves of the modelled PC population recreated the discriminability of comparison stimuli picked from different iso-feature-lines in the same sequence as observed with human psychometric curves. In the stimulus Range I the sequence was a→g→d and in Range II it was a→d→g (Fig. 5*C*,*D*; compare Fig. 4*D*,*G*). This relationship is predicted by the co-linearity of iso-feature-lines and iso-response line of PCs approximated in Figure 5*A*. In summary, the modeling exercise suggested that human performance on iso-feature-lines, as studied here, may be largely determined by PC population spiking activity.

**Figure 7.**
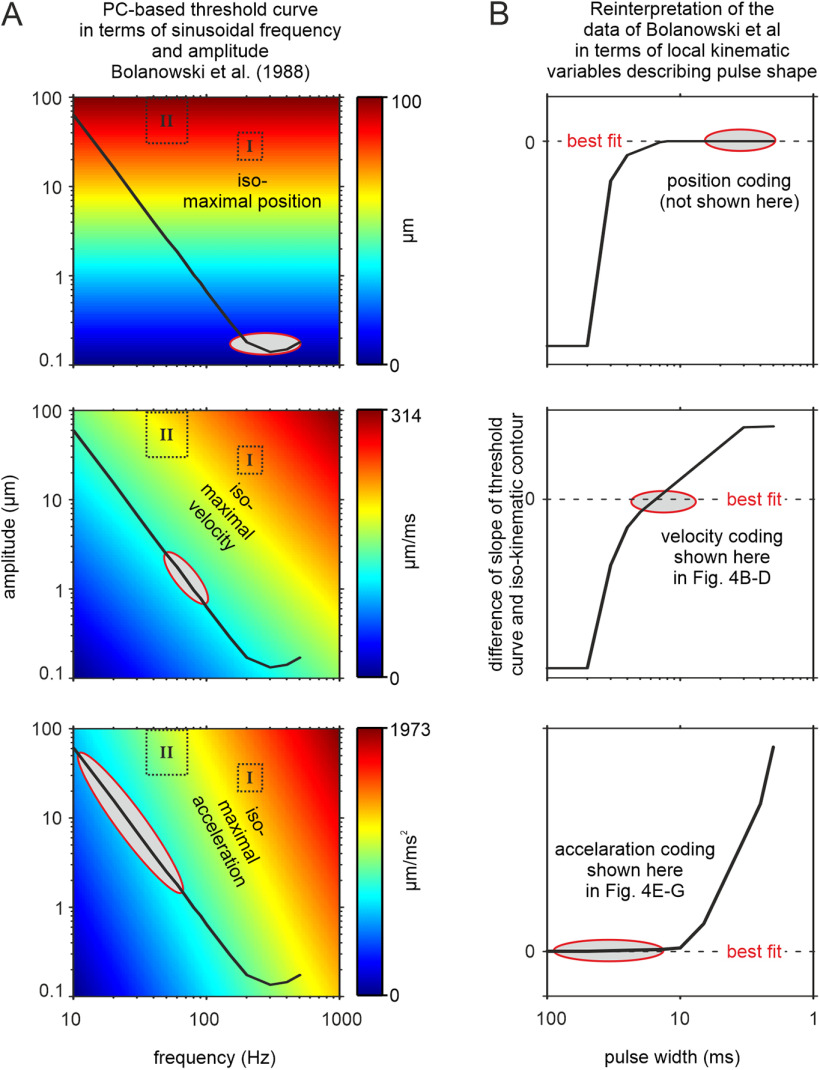
Reinterpretation of the classic sinusoidal frequency threshold curve for PCs, adapted from Bolanowski et al. (1988; see their Fig. 8), in terms of local coding variables (i.e., maximum position, maximum velocity, and maximum acceleration). ***A***, The threshold curve (black line) plotted across the original coordinates, sinusoidal frequency (abscissa) and amplitude (ordinate). The colored background in the three graphs codes for maximum position, velocity, and acceleration of the sinusoids. The stimulus Ranges I and II investigated in the present study (using sinusoidal pulses) are marked for reference (compare Fig. 4). Note that the color-coded iso-kinematic contours (i.e., the iso-feature-lines introduced in this study) appear as straight lines on the log-log scale. The ovals mark regions in stimulus space where kinematic variables vary little along the threshold curve (i.e., the slope of the PC-threshold curve and the slope of the iso-kinematic contour match). ***B***, Replotting the PC threshold curve in terms of the local kinematic variables. The abscissa is converted to pulse width (i.e., 1/f of the sinusoid) and the ordinate now holds the difference between the slope of the PC-based behavioral threshold and the slope of iso-kinematic contours. The best fit dashed lines and ovals indicate where the two slopes get very similar. These sites indicate stimulus sets that code optimally for the given kinematic variable, because the gradient (of the contours) orthogonal to the threshold curve is steepest. Conversely, stimuli located on or parallel to the kinematic contour (i.e., on the iso-feature-line) at these sites are difficult to discriminate, because PC activity and its concomitant perceptual effects do not vary. The behavioral data presented in Figure 4 and the modelled PC spike data in Figure 5 are compatible with this expectation that velocity coding is dominant at middle frequencies/pulse widths (close to 100 Hz/10 ms; Fig. 4*B–D*), while acceleration coding is optimal at low frequencies/narrow widths (>200 Hz/<5 ms; Fig. 4*E–G*).

Next, we aimed at recreating our experimental findings with mixed changes of pulse rate and shape as well using the model PC spiking. Again, by feeding the respective stimuli to the same model as before, we successfully recreated major characteristics of the psychophysical finding (Fig. 6). In this approach, we only modeled local coding (i.e., measuring spike responses to single pulses, typically consisting in 0 or 1 spike per pulse) because integrating the response across pulses rendered PC-based discriminability error-free (i.e., invariably reaching a discriminability value of 1), a phenomenon consistent with our notion (and with the results of numerous previous studies) that afferents are indeed temporally local encoders (Gerdjikov et al., 2010; Stüttgen and Schwarz, 2010; Birznieks et al., 2019). A trivial consequence of measuring responses to single pulses, from neurons devoid of spike adaptation, is that increasing pulse frequency does not affect discriminability. This is expressed by the coincident magenta and gray curves [Fig. 6, right; note, this is a discrepancy to the experimental data because human tactile system does integrate to a certain degree (compare Fig. 2*C*), very likely using neurons upstream of primary afferents]. The interesting part was thus modeling the second step of our experiment, compensating intensity increment (caused by additional pulses) by reduction of pulse amplitudes (red curve). Compared with the reference stimulus (magenta), reducing pulse amplitudes indeed increased discriminability by ∼0.2 (red), a fact that qualitatively matches human performance (compare Fig. 3*C*), and is compatible with the hypothesis that locally encoded information in primary afferent spikes is used as such, without relevant further integration, by the human tactile system up to the perceptual level.

## Discussion

Using change detection of pulsatile stimuli, we present evidence that humans are able to discriminate shapes of single pulses in an instantaneous, and thus temporally local fashion. Our results favor the hypothesis that tactile encoding of pulsatile stimuli is sensitive to the shape of pulses to an extent not thought before, and that encoding of pulse rate, a purely global variable and major candidate encoding variable discussed in classical work, contributes relatively little. Moreover, our results are difficult to reconcile with intensity playing a major role in discrimination of pulse series, another global variable, and major classical contender. Interestingly, discrimination performance was not based on a unique kinematic variable describing the pulse shape. Rather, the psychophysical performance was predicted by computer simulations incorporating classical threshold and entrainment curves of the population of a digit’s PC primary afferents (Freeman and Johnson, 1982; Saal et al., 2017). In fact, we found that classical primary afferent and behavioral threshold curves can be well explained within a framework of temporally local coding, simply by considering instantaneous kinematics of long sinusoids, the classical stimuli, rather than their frequency. Based on such reinterpretation of classical data, we suggest that PCs, and in fact the ascending human tactile system including the perceptual levels, encode indentation pulses in a temporally local fashion. Such local coding is surely not exclusive, but our results are compatible with the notion of a dominant effect on perception (with the qualification that we presented only analytical stimuli that are quite distinct from natural skin stimulation). The encoded kinematic variable is not unique. Rather, it seems to depend on pulse width, reflecting “acceleration” with slow, “velocity” for the middle, and “position” with the fastest pulses.

### Local coding

As the three coding symbols “global intensity,” global “rate,” and “local shape” (Fig. 1) are partially dependent on each other, our experimental logic relied on trading them against each other as done before in the rat whisker-related system (Waiblinger et al., 2015a,b). The central role is taken by intensity, which in Figure 1*A* is defined as the “mean signal.” We eliminate intensity from the analysis by plotting behavioral results across it, thus providing a common base, on which the effect of the other two variables, local shape and global rate, can be compared. However, we had to deal with the problem that the exact formulation of intensity is far from clear. The early finding that subjective intensity is not only dependent on the sine amplitude but also on the frequency (LaMotte and Mountcastle, 1975) gave rise to more general definitions that are applicable to random vibrotactile stimuli, like mean velocity (Arabzadeh et al., 2003; Gerdjikov et al., 2010), or “power” which is proportional to mean squared velocity (Hipp et al., 2006). Here, we exploited the delineation of a relevant stimulus range, marked by poor responses, to systematically investigate a more extensive and relevant selection of intensity formulations. The six intensity formulations chosen are the relevant ones because their variance within the poor-response stimulus range is small, i.e., their iso-feature-lines span (and quite evenly) cover that range (Fig. 2*B*). Based on this logic, other intensity formulations are rendered implausible to play a role, at least for this particular set of stimuli: for one, intensity formulations based on position traverse the first quadrant of the plot (characterized by increasing pulse amplitude and decreasing width). This quadrant gives rise to very high detection rates, making position-based intensity formulations unlikely candidates to affect perception. Similar reasoning applies to derivatives of position higher than acceleration (e.g., jerk, etc.) and the usage of higher powers (i.e., >3). Those iso-feature-lines are located in the lower left of the fourth quadrant, another high-performance region (Fig. 2*B*, the gray region, in which changes of pulse amplitudes dominate those of pulse widths). Finally, velocity or acceleration can be taken to powers picked from rational numbers before summing, and thus define additional, non-tested iso-feature-lines within the relevant stimulus range. However, the possibility that we missed outstandingly relevant intensity formulations is small because the coverage of the stimulus space by the iso-feature-lines investigated here is quite dense and exhaustive. From the participants’ performance on these iso lines, we have no reason to assume that their performance is mapped in grossly non-smooth ways across the stimulus range; therefore, it is likely that intensity formulations with rational powers would smoothly interpolate the results obtained by the six formulations picked here.

The exercise of scaling psychophysical data from separate shape and rate changing experiments down to these intensity formulations did not yield an unambiguous explanation in terms of intensity coding: the curves obtained from these experiments plotted on the exhaustive list of possible intensity formulations (discussed in the previous paragraph) never consistently matched (Fig. 2*D*). A first conclusion from this finding was that intensity coding did not play a dominant role in our experiments, and that the other two coding variables, shape and/or rate, must have played a significant role for the generation of responses. Secondly, minimum and maximum detection probabilities reached in shape-change experiments exceeded those observed with rate-change stimuli, suggesting that participants mastered the former at greater ease as compared to the latter, pointing to a dominance of local cues over rate cues.

One argument barring the conclusion of importance of local cues would be that the sessions probing the stimuli on different iso-feature-lines and the rate-change experiments were conducted in a block-wise (one session one stimulus set) fashion, and the participants, or rather their tactile systems, may have been adaptive enough to read out a different cue each session. This possibility was excluded by combining shape and rate changes in the same stimulus. As a starting point for this experiment, we chose stimuli located on the iso-feature-line for mean acceleration (line d) for the following reasons: first, possible experiments based on other iso-feature-lines are less meaningful, as the response on all other iso-feature-lines is much better and may offer little chance to measure potential perceptual effects of our intensity manipulations via shape and rate. Second, scrutinizing the stimuli for their kinematic content revealed that changing mean acceleration led to identical proportions of change in in the intensity formulation based on the two neighboring derivatives of position (mean velocity and mean jerk). Thus, a grossly different contribution of these alternative intensity formulations cannot explain the results. Our finding of a largely asymmetric perceptual effect of such intensity-balanced rate-increase and shape-decrease supports our conclusion that the local shape cue is unarguably important for perception.

### Role of primary afferent responses for tactile perception

We found that human change detection performance cannot be explained by one uniquely encoded kinematic variable. Instead, performance was qualitatively explained by spiking of modeled PC afferents. The principal finding that primary afferent spiking characteristics directly determine behavioral decisions has long been reported in classic studies comparing psychometric and neurometric curves (Talbot et al., 1968; Johansson et al., 1982; Bolanowski et al., 1988). However, these researchers interpreted their results uniquely in the framework of global coding: the expectation that the end-point of vibrotactile perception was either intensity or frequency led to the view that a sine wave is an entity, the neuronal responses to which had to be temporally integrated to feed to perception. A second important aspect of these works was that detection thresholds (not discrimination thresholds) were compared with primary afferent spiking. That is, the ability to discriminate minute difference in local shape as investigated in the current study was not directly addressed in the previous studies.

The present data support the notion that (1) local shape is used by the human tactile perceptual system; and (2) this information, at least for the stimuli used here, may be carried by PC spiking. We wish to emphasize that local coding is not mutually exclusive with global coding: in fact, we explicitly show that pulse rate changes, requiring purely global coding, can be perceived. Rather, we argue that local coding may come on top of global ones to improve perceptual capabilities. Based on our findings, we suggest a reinterpretation of the results of the classical studies to include aspects of local coding. We argue, firstly, that primary afferents are local encoders, i.e., in the framework of pulsatile stimuli, they use integration windows that are smaller than interpulse intervals. Thus, in the primary afferent’s view, a sine wave is not considered as an entity, but rather as a series of pulses to which spikes are generated according to instantaneous shape. This view is widely supported by the observation that myelinated primary afferents do not show considerable spike rate adaptation across repetitive stimuli of durations used here (i.e., <1 s; Freeman and Johnson, 1982; Whitsel et al., 2001; Deschênes et al., 2003; Leung et al., 2005; Bensmaïa et al., 2005; Chagas et al., 2013). Further, neuronal encoding studies failed in supporting the use of long integration windows (Vallbo et al., 1984; Jones et al., 2004; Chagas et al., 2013). Second, we argue that local shape information is preserved on ascending tactile pathways of rodents (Laturnus et al., 2021), a fact that may find its expression in the common finding of entrainment to repetitive events up to primate S1 (Mountcastle et al., 1969; Salinas et al., 2000). That way information of local shape may become accessible as well to the perceptual system. Moreover, neurometric performance based on decoding spike trains by integration is commonly found to be far superior than the actual psychophysical performance (Gerdjikov et al., 2010; Stüttgen and Schwarz, 2010; Birznieks et al., 2019), a finding at odds with the dominant use of spike integration as the basis for perception.

The schematic in Figure 7 attempts to visualize this reinterpretation using the PC-driven detection performance reported by Bolanowski et al. (1988; it is largely consistent with neurophysiological PC recordings by Freeman and Johnson, 1982). The threshold curve is typically rendered by using frequency of the sinusoidal stimulus mapped on the abscissa and its amplitude on the ordinate. We added information about the local variables, i.e., maximal position, velocity, and acceleration as color gradients, calculated from the sinusoidal waveform (Fig. 7*A*). In the local coding framework, the degree of co-linearity of the threshold curve with the contours of local kinematics would determine where a given local kinematic variable is expected to contribute the best as a viable cue. At sites of co-linearity, a certain stimulus deviation away from the threshold would fall on a steep gradient and thus would maximally engage primary afferent spiking. Inversely, changes in stimuli along or parallel to the threshold line would have minimal perceptual effect. We confirmed this effect by testing change detection (also known as discrimination) by picking stimuli from isolines (Fig. 4), while it was missed by the classical studies using detection psychophysics. Optimal alignment can be seen for maximal acceleration in the low frequency range; for velocity in the center range, and for position at high frequencies. In Figure 7*B*, we explicitly plot the change of local kinematics along the PC threshold curve. To do this we first translated the frequency axis to pulse width (i.e., wavelength; essentially reinterpreting the sinusoids used in the classical study as series of local pulses). The ordinate scales the difference of slope between threshold curve (= iso-response-line) and kinematic contours (= iso-feature-lines). The line labeled “best fit” indicates where this difference is zero, i.e., pointing to perfect alignment of threshold curve and kinematic contour (marked by ovals). Seen from a local coding perspective, the contribution of PC afferents can be termed “acceleration coding” at pulses wider than >14 ms, “velocity coding” around a pulse width of 10 ms, and finally “position coding” with very narrow pulses of widths smaller than 5 ms. Our results in the two subranges (Fig. 4) are consistent with the first two coding principles (we did not test the suggested position coding using the narrow extremes of pulse widths).

The predominant engagement of model PCs and qualitative fit with psychometrics does not come as a surprise as it is a common finding that PCs are the most sensitive afferents to indenting vibrotactile stimuli (Johansson, 1978) as well as to touch of more natural surfaces (Weber et al., 2013). It is not clear why SA1 and RA1 afferents did not recreate the behavioral results. The SA1 and RA1 end organs are closely related to papillary ridges (Cauna, 1954), and may be much more sensitive to tangential shear (Johansson and Westling, 1987; Delhaye et al., 2021) than to indentation, a question that needs to be further clarified using spike recordings in the future. We did not model the fourth class of human tactile skin receptors, the slowly adapting afferents of type 2 (Ruffini). They are thought to be specialized more to shear, and were shown to be less responsive to indentation stimuli (Johansson and Vallbo, 1979).

### What is the functional advantage of a local code?

The search for local coding is motivated by the assumed presence of frictional movements in objects that engage in moving contact (Schwarz, 2016). Moving contact is at the heart of tactile processing, as palpation movements are indispensable for the perception of fine textures (Hollins and Risner, 2000; Hollins et al., 2001; Skedung et al., 2013). Prototypical expressions of frictional contact are stick-slip events, short lived elastic deformations of the contacting materials coming about by sticking to surface elements, storing energy in elastic deformation, and releasing them quickly into sudden slips when frictional force is overcome by the driving movement of the contact (Schwarz, 2016). In the rodent vibrissa-based tactile system, frictional slips have been shown to exist (Arabzadeh et al., 2005; Ritt et al., 2008; Wolfe et al., 2008), and to encode texture information (Wolfe et al., 2008; Oladazimi et al., 2018). Primary sensory cortex in these animals has been shown to be selective for slip-based tactile inputs (Jadhav et al., 2009). Stick-slip events are short-lived, typically exceed noise in the signal, and therefore are accessible by local decoding schemes (Fig. 1*A*). In support of this notion, manipulations of pulsatile whisker deflections in a psychophysical study, have provided strong evidence in favor of local coding in rats’ tactile system (Waiblinger et al., 2015a). The present study extends this insight to the human fingertip system.

We wish to emphasize that the present study does not speak to the presence or absence of frictional movements or their use for perception in the human fingertip. However, it clearly provides supporting evidence for the perceptual hypothesis of temporally local stimulus read-out, a possible adaptation to make optimal use of information in frictional processes. Direct evidence about frictional movements of glabrous skin has so far been scant, but by no means negligible: papillary ridges undergo complex shearing deformations at the onset of a finger movement (Delhaye et al., 2016), and friction has been shown to be a determinant of roughness estimation, most of all for the discrimination of microscopic surface elements (Verrillo et al., 1999). Research on prehension has unearthed unequivocal evidence that sudden slip movement does occur in the skin, and is readily detected by humans to adjust grip forces (Johansson and Westling, 1987). Finally, so-called rate hardness (an instantaneous measure of change of force and speed when tapping surfaces) relates tightly to hardness perception suggesting that it may be based on local elements of the tactile signal as well (Lawrence et al., 2000; Han and Choi, 2010).

Our results are based on indentation stimuli, while frictional processes are often associated with tangential movement contact and ensuing skin shear (Johansson and Westling, 1987; Delhaye et al., 2014). In the whisker-related system latitudinal deflection, a sideways movement that can be compared with tangential skin movement, principally results also in longitudinal forces (along the whisker shaft), readily sensed by the neurites (Stüttgen et al., 2008; Quist et al., 2014; Oladazimi et al., 2021). We posit that papillary ridges activated by tangential skin movement would similarly generate force and moments that act in the direction normal to the surface of the skin. Moreover, real-world surfaces with asperities that stand out of the surface in 3D, are supposed to indent the skin locally, even if the main movement direction is tangential. In summary, the finding of local coding with indentation stimuli is expected as a partial requirement to assess frictional movement.

## Synthesis

Reviewing Editor: Karen Davis, Krembil Research Institute Decisions are customarily a result of the Reviewing Editor and the peer reviewers coming together and discussing their recommendations until a consensus is reached. When revisions are invited, a fact-based synthesis statement explaining their decision and outlining what is needed to prepare a revision will be listed below. The following reviewer(s) agreed to reveal their identity: Rochelle Ackerley.

The manuscript advances our understand of how small changes in touch input in humans can determine perception. This was further modeled via afferent simulations. The combination of these two approaches is highly complementary and the results show that humans readily use local coding information, which adds a lot to our understanding about how touch is processed.

The manuscript aims to investigate whether humans can use temporally local shapes of vibrotactile waveforms for tactile perception. Overall, the paper contains some difficult concepts but I think those concepts are reasonably well explained. The work has many implications on how we understand the precise tactile signals from the periphery. Overall, the area under investigation is interesting and the figures are very well presented. However, there are presentation issues and issues related to the computational modeling and the interpretation of those data that need to be addressed.

Major Comments:

1. The presentation of the study (methods and results) is somewhat confusing and difficult to follow, as there were various different experiments and ranges of stimuli, which are not well-explained. I have some further comments below, but I would urge the authors to reconsider how they structure their experiments, in terms of the layout (the figures very much help the reader understand).

2. In the introduction, the definition of local and global encoding in touch needs to be clarified more. This is a complicated concept and currently the text does not provide a clear-cut definition with examples of what exactly local and global encoding is, with examples of these. Figure 1 does help, although it may need some more explaining.

3. More background would be useful in the introduction, to set the scene for why the parameters here were chosen (e.g. frequencies).

4. In the methods:

a. the section on which subjects participated in which experiments it is a little complicated (just before the Vibrotactile stimulation part). This paragraph mentions different types of experiments that have not be covered before. It is difficult to know what the experiment actually was and how many subjects participated, it mentions pulse shape change with two ranges and also pulse rate change. I think the authors could insert a small section on the experimental design and what experiments were conducted on how many subjects. Also, if only 10 subjects participated in each part, was this sufficient?

b. To be clear, in the setup of the equipment, there was no force feedback and the stimulator was in constant contact with the skin throughout the whole experiment?

c. How were the parameters of the stimulation (width, frequency, amplitude, etc.) chosen? It would be good to have a little background to this in the introduction too. It is also not so clear exactly what happened in each experiment and it may be good to explicitly state this (e.g. a list or table that clearly details what was changed for each experiment).

d. Were the stimuli repeated? Due to the complex presentation of the methods, it is difficult to find this information and exactly what happened in each experiment. The authors may want to revise the whole methods section for clarity.

e. In the ‘Perceptual tasks and psychophysics procedure’ section, what exactly was the feedback the subject received? Was the feedback that their answer was correct or did it tell them information about the trial (change/no change)?

f. Why was white noise played only in experiments 2 and 3?

5. The results contain a lot of text that is not to the point and re-explains the introduction/methods, as well as discussing some of their results. It would be good if the authors could revisit the text to remove the redundancy and discussion throughout and this would make understanding the actual results easier.

6. Regarding the use of the finger skin model, this is a very interesting aspect, but it would be good to also mention that this model does not take into account slowly adapting type II afferents (Ruffini), although the authors may want to bring up in the discussion that this is probably not too bad, as it is not likely that SAIIs would encode the parameters that they are testing very well (i.e. they would encode tangential skin stretch better). However, I am a little surprised that the type I afferents in the model did not account for more of the results. Could the authors present this information and/or discuss why these did not match the psychophysical data? Was it something to do with their paradigm (e.g. vibration) or could it be a more general aspect?

7. Lines 243-244 and 514-516 report that simulated responses of SA1 and RA afferents did not match human performance and are therefore not reported. This requires more explanation. What is the basis for saying that human performance was not matched? I am specifically concerned that stimulus range II seems to sit at a frequency range where RA afferents are quite sensitive. My own bias is to suspect that co-activation of RA and PC afferents could be important for perception of certain frequencies. At the very least, showing or describing some "negative" data can help readers interpret what’s happening, rather than accepting at face value that RA’s and SA’s are not involved.

8. More generally, it’s not clear to me how the global spike count differs from the local spike response. The spike count across multiple pulses is ultimately the same thing as the probability of spiking in response to each pulse if there really is no adaptation on the timescales tested (i.e. the neuron responds to each pulse independently). Unless the pulse rate changes, I don’t see how the read-out matters in this context, but maybe I’m missing something.

Other Comments:

1. Lines 529-530. "In the stimulus range I, the sequence was a->g->d and in range II it was a->g->d." The second sequence should be a->d->g instead, right?

2. Lines 548-549 and Fig 6, "PC afferents showed a larger change of firing in compensated pulse shapes (red) than the change observed in uncompensated ones (gray and orange)." I found this quite confusing. First, the legend for Fig 6 provides little information, which means the reader must go back to earlier figures to identify the differently colored curves. Furthermore, I think it would be much clearer to explain that relative to the reference (orange), increasing the pulse rate had no effect on PC firing (gray) whereas changing the pulse shape increased PC firing (red). Is my summary correct? This highlights a more general point, which is that the writing is pretty clear in the first half of the Results, but it falls off in the second half enough to risk causing significant confusion because of the difficult concepts being addressed.

3. Following on the last point, I wonder if iso-lines (starting in Fig 2) should be referred to as iso-feature lines, or something that specifies that one or another stimulus metric is the same across the parameter combinations shown on the x- and y-axes. When iso-response lines are introduced in connection with the model, there is room for confusion as to what iso-lines refers to (e.g. lines 504 and 531).

4. Fig 5B, the reference to pale and dark colors presumably relates to the drawing of the hand. This is too small to see.

5. Figure 7, colors seem to be unevenly distributed such that certain ranges (e.g. green) are very thick whereas others (e.g. yellow) are very thin. Is this just a display issue? I think the metrics depicted in color (max position, velocity and acceleration) vary smoothly, but one can get a different impression from the color. If I’m correct, the color transitions should be spaced more evenly. At the very least, a color scale bar should be provided.

6. In Figure 6, neither the tuning curve nor the iso-lines for max velocity or acceleration seem to bend in the frequency range below 100 Hz, so I do not understand the rational for drawing two separate ovals (i.e middle vs bottom panels). Please clarify

7. line 555, It is not obvious what "system" and "analysis" refer to. I think what the authors are trying to explain is that the action potential initiation process (or encoding more generally) is sensitive to the waveform of individual pulses, not just to the rate at which those pulses are repeated.

8. Line 655, the authors write that primary afferents "do not integrate the stimulus across stimulus time". I think the real issue is a quantitative one rather than a qualitative one; in other words, the integration time is shorter than the inter-pulse interval. The transduction/spike initiation process still takes time (i.e. integration still occurs) but it occurs so quickly that the process is local (i.e. the response to each pulse is independent from previous pulses). Elsewhere (e.g. line 671) the term spike integration is used in a way that refers to decoding (how does the neural activity produce sensation) rather than encoding (how do the vibrotactile stimulus produce neural activity). Both processes are important, but the ambiguous use of certain terms leaves room for confusion.

9. Line 667, I do not think that an individual spike can produce a sensation of vibration, so I would tend to leave out the Valbo reference.

10. As a minor point, I do not think the last paragraph is needed, as it simply summarizes ideas in the work. Rather, the end of the introduction would benefit from a short paragraph that clearly states the aims and hypotheses of the work.

11. ‘psychometric’ (psychological assessment) may be better replaced by ‘psychophysical’

12. Please avoid using terms like ‘We demonstrate for the first time’ (significance statement), as one never knows if something has been done before and in eNeuro, the aim is more ‘did we learn something useful or important?’ rather than novelty.
