# Supplementary figures and images for: Humans Use a Temporally Local Code for Vibrotactile Perception
Source: eNeuro. 2021 Nov 3;8(6):ENEURO.0263-21.2021. doi: 10.1523/ENEURO.0263-21.2021 (PMC8570683; doi:10.1523/ENEURO.0263-21.2021)

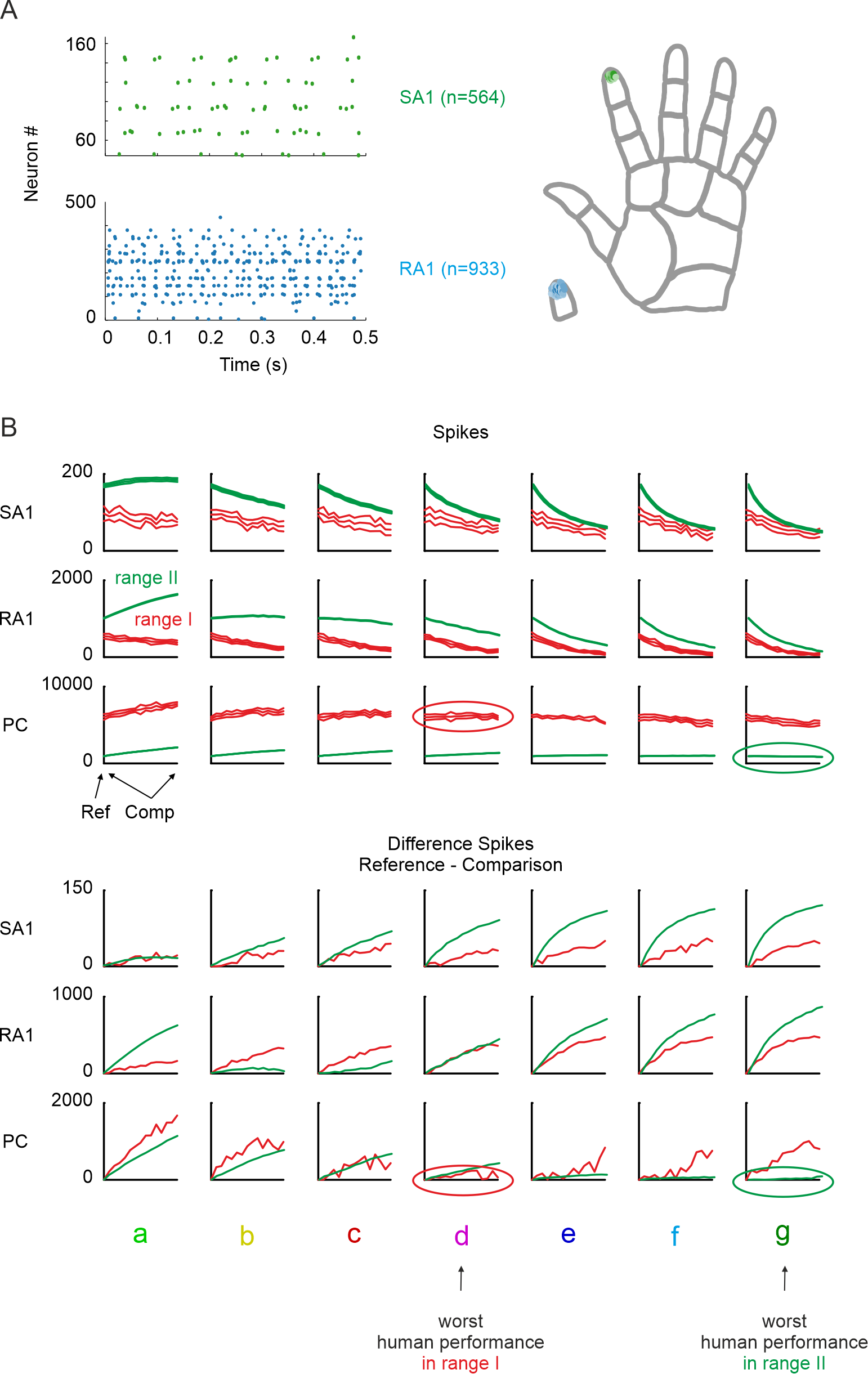

Supplement: Extended Data Figure 5-1 — Population spike responses of all three modelled primary afferent classes. A, The model contained 564 slowly adapting primary afferents type 1 (SA1, green), and 933 rapidly adapting primary afferents of type 1 (RA1, blue), all located close to the indented site on the fingertip (conventions of the hand drawing as in Fig. 5, for RA1 only the distal phalanx of the index finger is shown). Spiking in response to one trial (reference stimulus of Range I, duration 500 ms) is presented as raster plots. Each line represents the spiking of one modelled neuron; note that a majority of neurons did not spike at all to this stimulus (for PC data see Fig. 5). B, Summary response profiles for all three modelled primary afferent populations (rows of graphs). Each graph plots on the ordinate either the spike numbers (top, mean ± SD) or the mean absolute difference spike numbers between reference and comparison stimulus (bottom), summed across the neuronal population. The abscissae hold the reference stimulus (leftmost data point) as well as all comparison stimuli (to the right with decreasing pulse width). Red color labels data from Range I, green indicates data from Range II. Each column of graphs presents the responses to one iso-feature-line (labeled as in Fig. 2). The iso-feature-lines yielding worst population performances of participants for the two ranges (d, g) are marked at the bottom (compare Fig. 4). The ovals mark the indiscriminate responses of the PC population across reference and comparison stimuli to these iso-feature-lines. SA1 and RA1 afferents show indiscriminate responses to stimuli on line a (SA1) and b (RA1), and thus, by themselves, are unable to explain human performance. Download Figure 5-1, TIF file. [file enu-eN-NWR-0263-21-s03.tif]
